# Proanthocyanidin Synthesis in Chinese Bayberry (*Myrica rubra* Sieb. et Zucc.) Fruits

**DOI:** 10.3389/fpls.2018.00212

**Published:** 2018-02-28

**Authors:** Liyu Shi, Shifeng Cao, Xin Chen, Wei Chen, Yonghua Zheng, Zhenfeng Yang

**Affiliations:** ^1^College of Food Science and Technology, Nanjing Agricultural University, Nanjing, China; ^2^College of Biological and Environmental Sciences, Zhejiang Wanli University, Ningbo, China

**Keywords:** Chinese bayberry, proanthocyanidin, flavan-3-ols, anthocyanidin reductase, leucoanthocyanidin reductase

## Abstract

Proanthocyanidins (PAs) are distributed widely in Chinese bayberry fruit and have been associated with human health benefits, but molecular and biochemical characterization of PA biosynthesis remains unclear. Here, two genes encoding key PA biosynthetic enzymes, anthocyanidin reductase (ANR) and leucoanthocyanidin reductase (LAR) were isolated in bayberry fruit. *MrANR* was highly expressed at the early stage of fruit development when soluble PAs accumulated at high levels. Meanwhile, the transcript abundance of both *MrANR* and *MrLAR* observed at the late stage was paralleled with the high amounts of insoluble PAs. LC-MS/MS showed that PAs in developing Chinese bayberry fruits were comprised predominantly of epigallocatechin-3-O-gallate terminal subunits, while the extension subunits were a mixture of epigallocatechin-3-O-gallate, epigallocatechin and catechin. Recombinant MrANR protein converted cyanidin to a mixture of epicatechin and catechin, and delphinidin to a mixture of epigallocatechin and gallocatechin *in vitro*. Recombinant MrLAR was active with leucocyanidin as substrate to produce catechin. Ectopic expression of *MrANR* in tobacco reduced anthocyanin levels but increased PA accumulation. The catechin and epicatechin contents in transgenic flowers overexpressed *MrANR* were significantly higher than those of wild-type. However, overexpression of *MrLAR* in tobacco led to an increase in catechin levels but had no impact on PA contents. Quantitative real time PCR revealed that the loss of anthocyanin in transgenic flowers overexpressed *MrANR* or *MrLAR* is probably attributed to decreased expression of tobacco chalcone isomerase (CHI) gene. Our results not only reveal *in vivo and in vitro* functions for ANR and LAR but also provide a resource for understanding the mechanism of PA biosynthesis in Chinese bayberry fruit.

## Introduction

Several different classes of metabolites are synthesized via the flavonoid pathway, including anthocyanins, proanthocyanidins (PAs) and flavonols, which occur in a wide range of plants and play important roles during plant development (Winkel-Shirley, 2001). PAs, also known as condensed tannins, are involved in plant defenses against pathogens and herbivores due to their ability to bind proteins and metal ions (de Colmenares et al., 1998; Dixon et al., 2005). It is well documented that PAs can affect flavor and astringency in fruits, fruit juices, tea, or wine and protect ruminants from potentially pasture bloat (McMahon et al., 2000; Aron and Kennedy, 2008). Furthermore, PAs are beneficial to human health by protecting against cardiovascular disease, cancer establishment and progression, and bacterial infections (Bagchi et al., 2000; Cos et al., 2004; Dixon et al., 2005).

**Figure 1:**
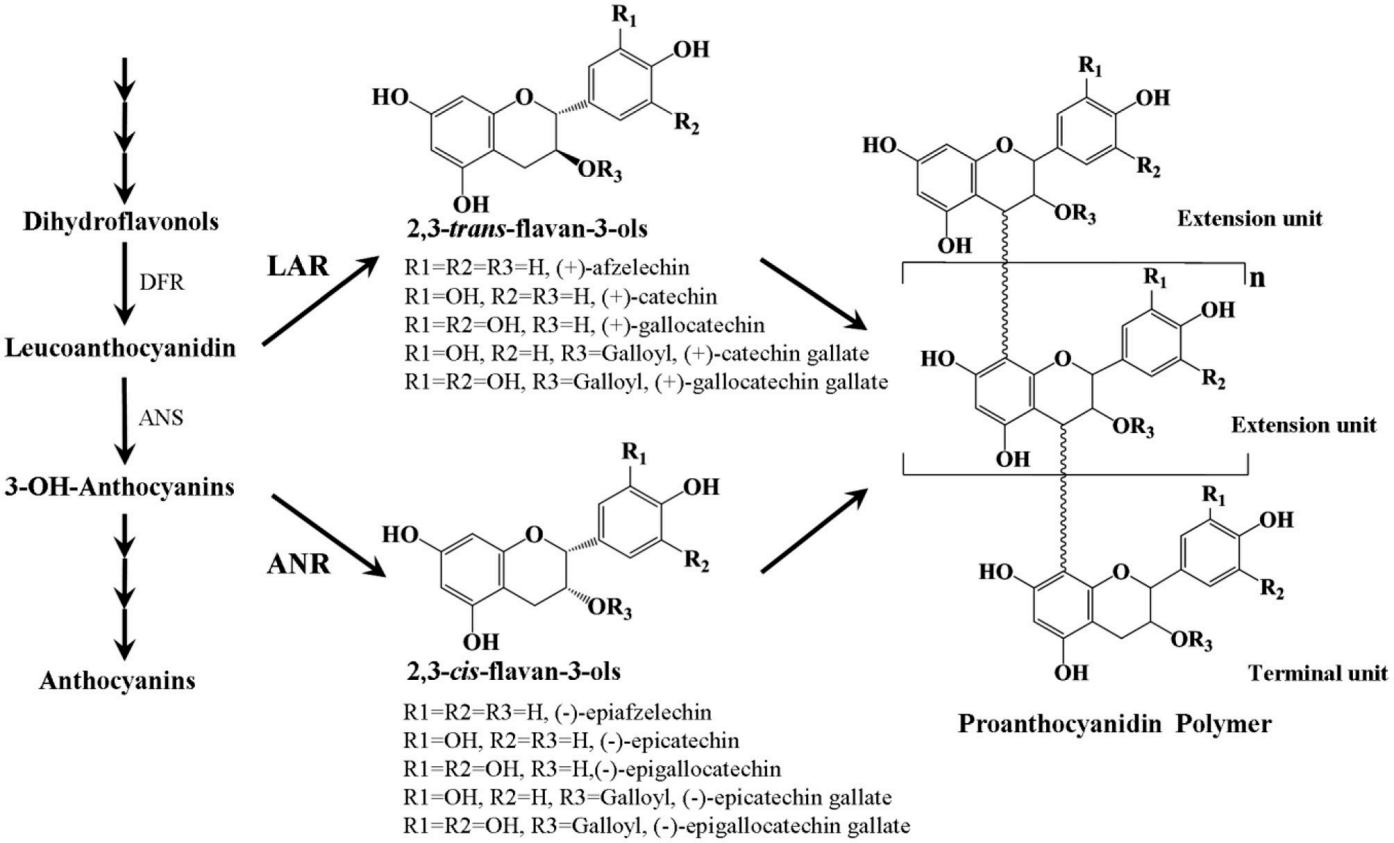
Scheme of the proanthocyanidin synthesis pathway. DFR, dihydroflavonal 4-reductase; ANS, anthocyanidin synthase; LAR, leucoanthocyanidin reductase; ANR, anthocyanidin reductase.

PAs are colorless flavonoid polymers formed by polymerization of flavan-3-ol units and are synthesized via the pathway leading to the synthesis of anthocyanins (Figure 1, Lepiniec et al., 2006). The key enzymes involved in PAs biosynthesis are leucoanthocyanidin reductase (LAR) and anthocyanidin reductase (ANR), which act in the production of 2,3-trans-flavan-3-ols [(+)-afzelechin, (+)-catechin, and (+)-gallocatechin] and 2,3-cis-flavan-3-ols [(−)-epiafzelechin, (−)-epicatechin, and (−)-epigallocatechin], respectively (Tanner et al., 2003; Xie et al., 2003). Although 2,3-trans- and 2,3-cis-flavan-3-ols only differ by the stereochemical configuration at the C2 and C3 positions, they can be identified by HPLC and LC-MS/MS (Zifkin et al., 2012; Ferraro et al., 2014). Quite commonly, these flavan-3-ol monomers can be esterified with gallic acid. Some gallate ester flavan-3-ols, such as (−)-epicatechin-gallate and (−)-epigallocatechin-gallate have been shown to be major phenolic compounds in persimmon (Gu et al., 2008), tea (Molan et al., 2009), and grape (Xie and Dixon, 2005). Both *ANR* and *LAR* genes have been characterized from several plant species, except for Arabidopsis, which lacks an intact LAR orthologue. In Arabidopsis, expression of the *ANR* gene is limited to the seed coat (Devic et al., 1999), while in many other plants such as grape (Bogs et al., 2005), Medicago (Pang et al., 2007), and cacao (Liu et al., 2013), expression of both *ANR* and *LAR* genes was observed in a range of different tissues including seeds, leaves, and flowers. In addition, it was reported that overexpression of cacao *TcANR* in Arabidopsis banyuls mutants which accumulate anthocyanins, instead of colorless proanthocyanidin precursors complemented the PA deficient phenotype of the seed coat (Liu et al., 2013). Ectopic expression of *LAR* genes from Medicago (Pang et al., 2007) or cacao (Liu et al., 2013) and *ANR* genes from apple (Han et al., 2012) or grape (Bogs et al., 2005) in tobacco petals resulted in accumulation of PAs but reduced anthocyanidin levels. However, a loss of PA accumulation was also observed in flowers of transgenic tobacco lines overexpressing the apple *MdLAR1* gene (Liao et al., 2015).

Chinese bayberry (*Myrica rubra* Sieb. et Zucc.) is an important subtropical fruit crop, which is originated in eastern Asia, mainly in China. Recently, it has been received much attention due to its high levels of flavonoids, which is believed to provide benefits to human health (Bao et al., 2005). Since fruits are important edible source of PAs, many studies have focused on the PA synthesis of fruits, such as grape (Bogs et al., 2005), apple (Han et al., 2012; Liao et al., 2015), persimmon (Akagi et al., 2009), blueberry (Zifkin et al., 2012), and strawberry (Almeida et al., 2007). In these fruits, both *ANR* and *LAR* genes contribute to PA synthesis and the accumulation of PAs varies in different species and at different developmental stages of the fruit. Recently, PAs in Chinese bayberry leaves were first qualitatively analyzed by Fu et al. (2014). However, the PAs accumulation and biosynthesis in Chinese bayberry fruit is still unknown.

In this study, we measured PA content and chemically characterized the PAs in Chinese bayberry fruit during ripening. The expression profiles and functional characterization of *MrANR* and *MrLAR* in relation to PA biosynthesis were investigated. The results presented here further our understanding of the molecular mechanism of the PA biosynthesis in Chinese bayberry.

## Materials and methods

### Plant material

Bayberries (*Myrica rubra* Sieb. and Zucc. cv. Biqi) used in this study were obtained from five adult trees cultivated in a homogeneous orchard in Cixi, Zhejiang Province. Fruit were hand-harvested at five developmental stages from 57 to 113 days after flower bloom (DAFB) with an interval of 2 weeks. At each stage, 15 fruit picked from each bayberry trees were collected. All samples were transported to the laboratory within 1 h and the fruit flesh of each whole fruit were immediately frozen in liquid nitrogen, ground to a fine powder and then stored at −81°C until analyzed.

Tobacco plants (*Nicotiana tabacum* var. Samsun) were cultured in a greenhouse at 25°C under 16 h/8 h light-dark illumination.

### Analysis for PA contents and composition

For extraction of PAs, approximately 0.2–0.5 g of grounded tissues (bayberry fruit flesh or tobacco petals) were extracted with 5 mL of extraction solution (70% acetone: 29.5% water: 0.5% acetic acid) by vortexing for 30 s followed by sonication at 30°C for 15 min. After centrifuging at 13,000 g for 10 min, the pellet was re-extracted twice as above. Pooled supernatants were then extracted twice with chloroform and three times with hexane.

The PA subunit composition was analyzed using LC-MS/MS following acid-catalyzed cleavage in the presence of excess phloroglucinol. For phloroglucinol degradation of the PAs, the method of Cavallini et al. (2015) was used with some modifications. In brief, a 400 μL aliquot of supernatant was freeze-dried overnight and dissolved in 100 μL of reagent solution [0.25 g of phloroglucinol, 0.05 g of ascorbic acid, and 5 mL of acidified methanol (0.2 N HCl)]. The mixture was heated at 50°C for 100 min and then 100 μL of sodium acetate buffer (200 mM; pH 7.5) was added to quench the reaction. After centrifugation for 15 min at 13,000 g, 20 μL of supernatant was run on a Waters ACQUITY UPLC system (Milford, MA, USA) equipped with an ACQUITY UPLC photodiode array detector (Waters). Molecules were separated on a Sunfire C18 column (4.6 × 150 mm, 5 μm particle size) with a pre-column of the same material (4.6 × 10 mm, 4 μm particle size) (Waters) at 25°C and at a flow rate of 0.7 mL min^−1^. Elution was performed with 0.2% (v/v) aqueous formic acid (solvent A) and methanol with 0.2% formic acid (v/v; solvent B), using the following elution program: initially 5% B for 4 min; 67% B at 25 min; 90% B at 25.5 min; and isocratic at 90% B from 25.5 to 30 min. All phloroglucinol adducts of flavan-3-ols and flavan-3-ol terminal units were monitored at 280 nm. Mass spectrometry was carried out using a Waters Q-TOF Synapt G2 mass spectrometer. The mass spectrometer conditions used were as follows: capillary voltage, 2200 V; cone voltage, 40 V; extractor voltage, 4 V; source temperature, 105°C; desolvation temperature 350°C; cone gas flow (N_2_), 50 L h^−1^ and desolvation gas flow (N_2_), 800 L h^−1^. The spectra ranging from m/z 50 to 1,200 were taken in the negative mode. The energies for collision-induced dissociation were 20–40 V for fragmentation. The data were accumulated for 0.2 s per cycle and calculated using the MassLynx 4.1 software (Waters). PA terminal subunits levels were calculated by comparison with commercial standards. The concentration of extension subunit-phloroglucinol adducts was determined using published molar extinction coefficients (Kennedy and Jones, 2001; Zhang et al., 2016). All compounds were expressed as catechin equivalents. The mean degree of polymerization (mDP) was calculated from the molar ratio of all subunits (terminal and extension subunits) to terminal units.

Total soluble PA content was determined spectrophoto-metrically after reaction with dimethylaminocinnamaldehyde (DMACA) reagent (0.2% [w/v] DMACA in methanol-3 N HCl [1:1]) at 640 nm (Pang et al., 2007). The residues from the above tissue extractions were used for insoluble PAs analysis using the method of Pang et al. (2007). Total soluble PA or insoluble PA levels were calculated as procyanidin B2 equivalents.

### Anthocyanin analysis

The total anthocyanin content of tobacco petals was determined according to the method described by our group (Shi et al., 2014), which involves measuring the absorbance (510 and 700 nm) of extracts that have been diluted with pH 1.0 and 4.5 buffers.

### RNA extraction and cDNA preparation

Total RNA was isolated from bayberry fruit flesh and tobacco petals using a Plant RNA Kit (Omega, Norcross, GA) according to the manufacturer's instructions. DNA contamination was removed from RNA preparations using RNase-free DNase I (Omega, Norcross, GA). Approximately 2 μg of total RNA was reverse-transcribed with the SuperRT First Strand cDNA Synthesis Kit (CWBIO, Beijing, China), following the manufacturer's instructions.

### Real-time quantitative RT-PCR

Real-time Quantitative RT-PCR (qRT-PCR) analysis was performed on a Bio-Rad CFX96 Real-Time PCR System (Bio-Rad, Hercules, CA, USA) using LightCycler 480 SYBR Green Master (Roche, Shanghai, China) following the manufacturer's instructions. Each reaction contained 0.5 μL of template cDNA, 0.5 μL of 10 mM forward and reverse primers, 6.25 μL of SYBR Green PCR Master Mix, and 4.75 μL of RNase-free water in a total volume of 12.5 μL. The transcript levels were normalized to *MrACT* (GenBank accession no. GQ340770) to minimize variation in cDNA template levels. All gene expression analyses were performed with three independent biological replicates, and primer sequences used for qRT-PCR are listed in Table S1.

### Cloning of *MrANR* and *MrLAR*

Partial sequences of *MrANR* and *MrLAR* were isolated using degenerate primers (Table S1) designed from conserved regions of corresponding genes reported from other species by the CODEHOP strategy (Rose et al., 1998). The 5′ and 3′ ends of the coding sequence were obtained by rapid amplification of cDNA ends (RACE) using the SMART RACE cDNA amplification kit (Clontech, Mountain View, CA, USA). PCR fragments were subsequently cloned into pMD18-T vector (Takara, Japan) and sequenced. The complete open reading frames (ORFs) of *MrANR* and *MrLAR* were amplified from bayberry (Biqi) cDNA derived from fruit sampled at 113 DAFB PCR products were then ligated into the pMD18-T vector (Takara, Japan) and subjected to DNA sequencing. The primers used are shown in Table S1.

### Expression and purification of recombinant *MrANR* and *MrLAR*

The ORFs of *MrANR* and *MrLAR* were amplified from Biqi fruit (113 DAFB). The PCR product was purified and subcloned into the *Bam*HI and *Sal*I sites of the pET32a expression vector (Novagen, Gibbstown, NJ, USA). After confirmation by sequencing, the recombinant vectors were introduced into *Escherichia coli* BL21 (TransGen, Beijing, China). The bacteria were grown in Luria-Bertani ampicillin (100 μg mL^−1^) medium at 37°C overnight until the OD600 reached 0.6, at which point 1 mM IPTG (isopropyl β-D-1-thiogalactopyranoside) was added to induce protein expression at 120 rpm for 48 h at 16°C. The recombinant proteins with a 6 × His tag at the N terminus were purified with the His-tagged protein purification kit (CWBIO, Beijing, China), according to the manufacturer's instructions. Purity was then checked by SDS-PAGE.

### Assay of ANR and LAR activities

3,4-*cis*-Leucocyanidin was prepared by reduction of (+)-dihydroquercetin with sodium borohydride, modified from the method of Tanner and Kristiansen (1993). Briefly, a solution containing 2 mg (+)-dihydroquercetin in 250 μL of dry ethanol was added to 0.2 mg of solid sodium borohydride and incubated at 20°C for 2 h. After that, 5 ml of 0.1% (v/v) acetic acid was added into the mixture and incubation for 4 h at 40°C. The epimerization was halted by freezing in liquid nitrogen after 3 h and lyophilization. LAR activity for production of catechin from 3,4-*cis*-leucocyanidin was determined in a final volume of 200 μL containing 100 mM Tris-HCl (pH 7.5), 1 mM NADPH, 0.1 mM 3,4-cis-leucocyanidin, and 1 mg of recombinant MrLAR protein. For assay of recombinant MrANR with cyanidin or delphinidin as substrate, reaction mixtures (200 μL) contained 100 mM Tris-HCl (pH 6.0), 1 mM NADPH, 0.1 mM cyanidin or delphinidin, and 1 mg of recombinant MrANR protein. After incubation at 30°C for 30 min, the reaction was terminated by extracting twice with 400 μL ethyl acetate, vortexing for 1 min, and centrifuging for 1 min. The solvent was evaporated, and the organic fraction was resuspended in 200 μL of methanol and then subjected to LC-MS/MS analysis. Crude protein extract from induced BL21 containing empty vector was used as a control.

LC-MS/MS analysis of enzymatic products was performed using a Waters ACQUITY UPLC system (Milford, MA, USA) equipped with an ACQUITY UPLC HSS T3 column (2.1 × 100 mm, 1.8 μm particle size; Waters) at a flow rate of 0.2 mL min^−1^ and a column temperature of 25°C. The mobile phase consisted of 0.2% acetic acid (solvent A) and methanol (solvent B). The gradient conditions were: 0 min, 5% B; 15 min, 70% B; 15.2 min, 95% B; 16.2 min, 95% B and 16.4 min, 5% B. The mass spectrometer conditions were the same as for the PA subunit composition analysis.

### Transformation of tobacco

The coding sequences of *MrANR* and *MrLAR* were amplified by PCR, and cloned into the pCAMBIA-1300 binary vector (CAMBIA, Canberra, Australia) using the *Bam*HI and *Sal*I sites. The resulting binary vectors were transferred into Agrobacterium tumefaciens strain EHA105 by electroporation. Agrobacterium-mediated transformation in tobacco was carried out using the leaf disk method (Horsch et al., 1989). Transgenic plants were selected on Murashige and Skoog medium supplemented with 25 mg /L hygromycin.

## Results

### Molecular cloning and sequence analysis of *MrANR* and *MrLAR* genes

A full-length cDNA of *MrANR* was isolated by 5′- and 3′-RACE; the cDNA was 1355 bp long and had an ORF of 1014 bp encoding a 337-amino acids protein. A BLASTP search revealed that the deduced amino acid sequence was 82% identical with *ANR*s from *Vitis vinifera* (*VvANR*), *Theobroma cacao* (*TcANR*), or *Malus domestica* (*MdANR*). Multiple sequence alignment (Figure S1) showed that *MrANR* contained a Rossmann dinucleotide-binding domain (motif GXGXXA) (Bottoms et al., 2002), which is conserved among ANR proteins (Greco et al., 2012). The calculated pI and molecular mass of the deduced MrANR protein were 6.36 and 36 kDa, respectively.

The *MrLAR* cDNA was 1531 bp in length and contained an ORF of 1083 bp encoding a protein of 360 amino acids with a predicted pI of 6.06 and a molecular mass of 39 kDa. *MrLAR* at the amino acid level showed 77% identity to *CsLAR* (*Camellia sinensis*), 72% to *TcLAR* (*Theobroma cacao*), and 70% to *VvLAR* (*Vitis vinifera*). The MrLAR protein contained the LAR characteristic amino acid motifs such as RFLP, ICCN, and THD (Tanner et al., 2003; Bogs et al., 2005; Figure S2).

### Expression of *MrANR* and *MrLAR* during fruit development

**Figure 2:**
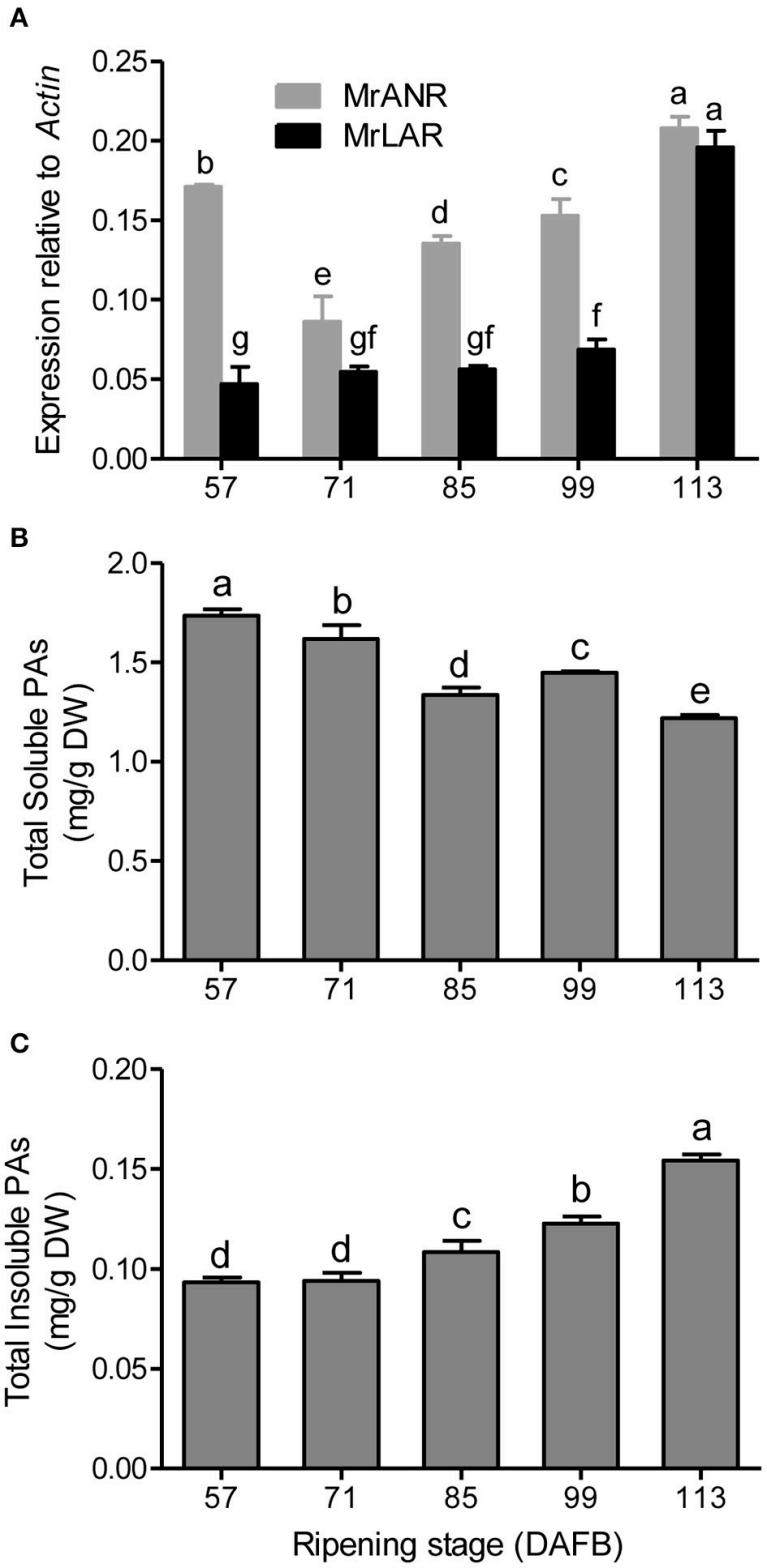
PA accumulation and gene expression of *MrANR* and *MrLAR* during ripening in Chinese bayberry fruit. **(A)** Relative transcript levels of of *MrANR* and *MrLAR*. Levels of soluble PAs **(B)** and insoluble PAs **(C)**. Different letters above the bars indicate significant differences among different stages (*P* < 0.05 by Duncan's multiple range test).

The transcript abundance of *MrANR* was at a high level at 57 DAFB, then declined at 71 DAFB followed by an increase toward to maturity (Figure 2A). Expression of *MrLAR* remained relatively low from 57 to 99 DAFB, but a significant increase was observed at 113 DAFB (Figure 2A).

### PA accumulation in developing bayberry fruit

To understand temporal PA accumulation in Chinese bayberry fruit, levels of total soluble and insoluble PAs at different developmental stages were measured (Figures 2B,C). The concentration of soluble PAs was highest in fruit at 57 DAFB, and declined thereafter. No significant changes in the levels of insoluble PAs were observed at the early developmental stages (57 to 71 DAFB) but the content increased to a maximum at 113 DAFB.

**Figure 3:**
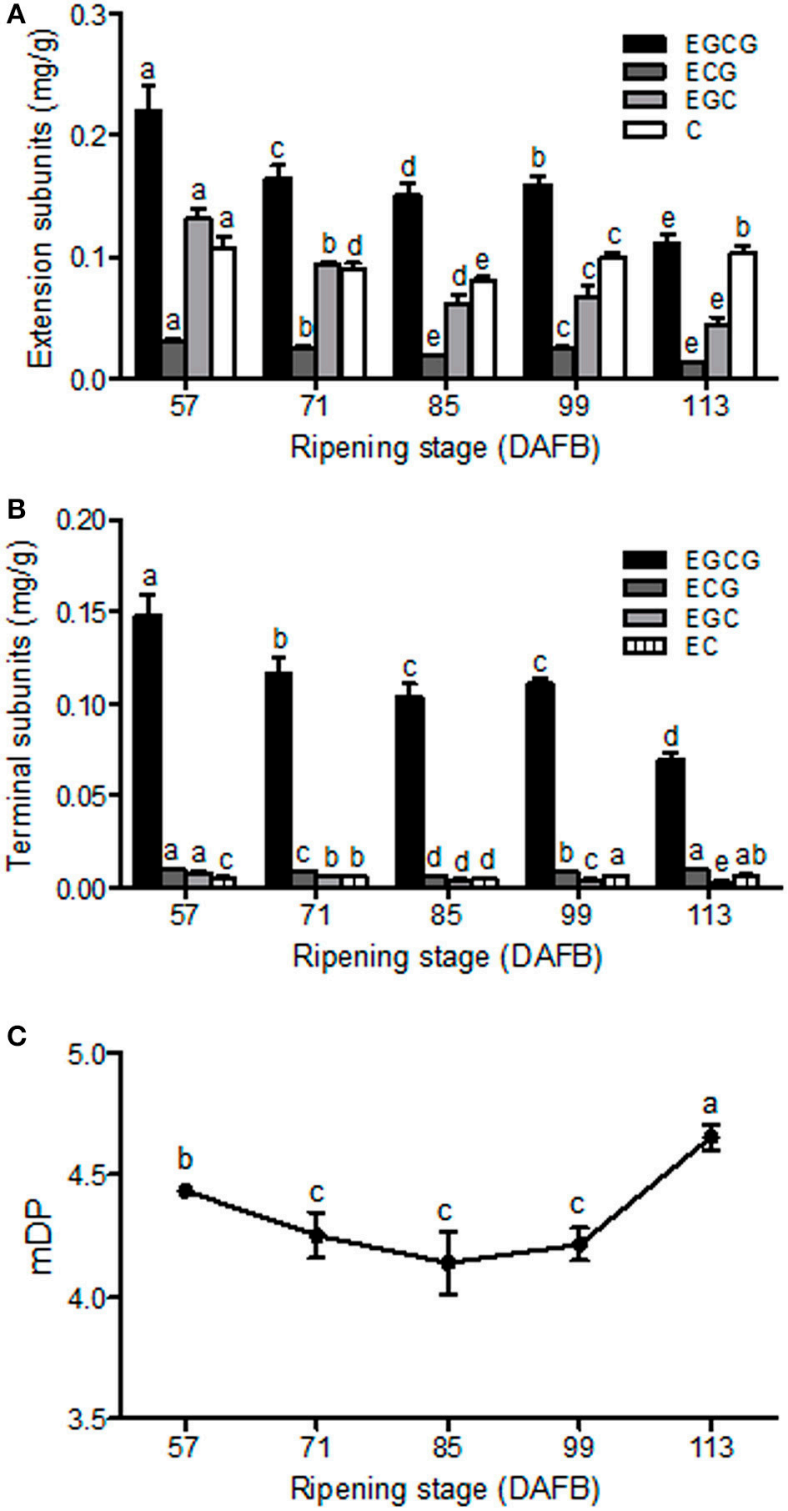
PA composition in developing Chinese bayberry fruit. Accumulation and composition of PA extension subunits **(A)** and terminal subunits **(B)**. EGCG, epigallocatechin-3-O-gallate; ECG, epicatechin-3-O-gallate; EGC, epigallocatechin; EC, epicatechin; C, catechin. **(C)** Mean degree of polymerization of PAs in developing bayberry fruit from 57 to 113 DAFB. Different letters above the bars indicate significant differences among different stages (*P* < 0.05 by Duncan's multiple range test).

To further analysis the structure of Chinese bayberry fruit PAs, the soluble PA oligomers was subsequently subjected to acid-catalyzed cleavage in the presence of phloroglucinol. The released phloroglucinol adducts (extension units) and terminal flavan-3-ol units were then identified and quantified by LC-MS/MS (Table S2 and Figures 3A,B). The PA extension subunits were consisted mostly of epigallocatechin-3-O-gallate (EGCG), epigallocatechin (EGC) and catechin (C), with much lower levels of epicatechin-3-O-gallate (ECG) units. The concentration of EGCG, EGC and ECG extension subunits steadily decreased until fruit maturation, while a small increase in catechin occurred at 99 DAFB (Figure 3A). For flavan-3-ols, EGCG was the predominant terminal subunit detected in fruit at all stages (78–88%) and displayed a decline in content over development (Figure 3B). Minor amounts of ECG, EGC and epicatechin (EC) were also detected in the PA terminal subunits, but only the EGC content decreased during ripening (Figure 3B). In developing bayberries, the mDP of the PA polymers was around 4.5 (Figure 3C).

### Functional analysis of recombinant *MrANR* and *MrLAR in vitro*

**Figure 4:**
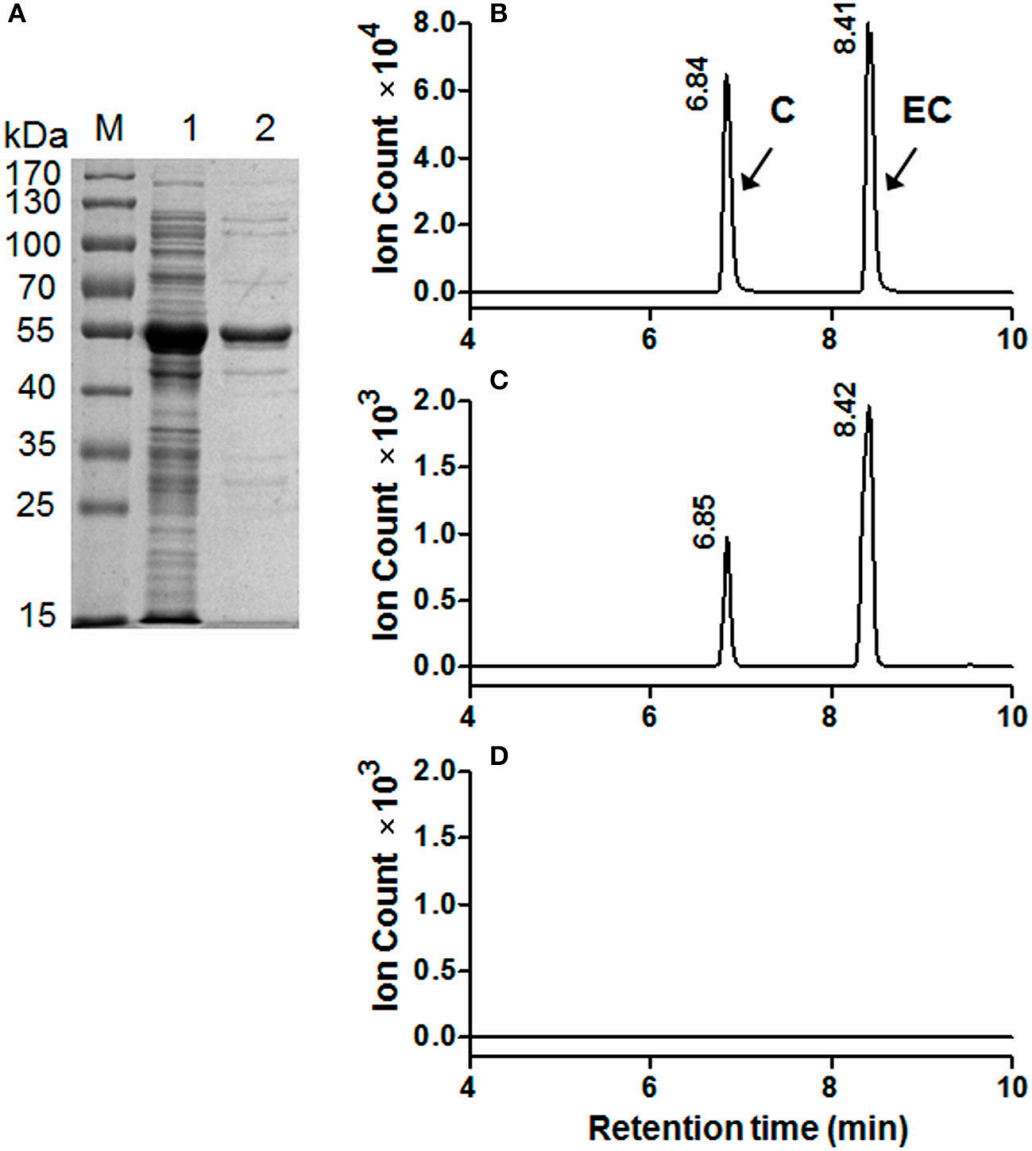
Enzymatic assay of recombinant MrANR using cyanidin as a substrate. **(A)** Analysis of MrANR protein on a 12.5% SDS-PAGE gel. M, Molecular weight marker; 1, crude protein from *E. coli* BL21 (DE3) harboring pET32α-MrANR induced by IPTG; 2, purified recombinant MrANR protein. **(B–D)**: LC-MS [M-H]^−^ extracted ion chromatographs (*m*/*z* = 289) of authentic (+)-catechin **(C)** and (−)-epicatechin (EC) **(B)** along with the enzymatic products of purified recombinant MrANR protein **(C)** or protein extract from vector control **(D)**.

**Figure 5:**
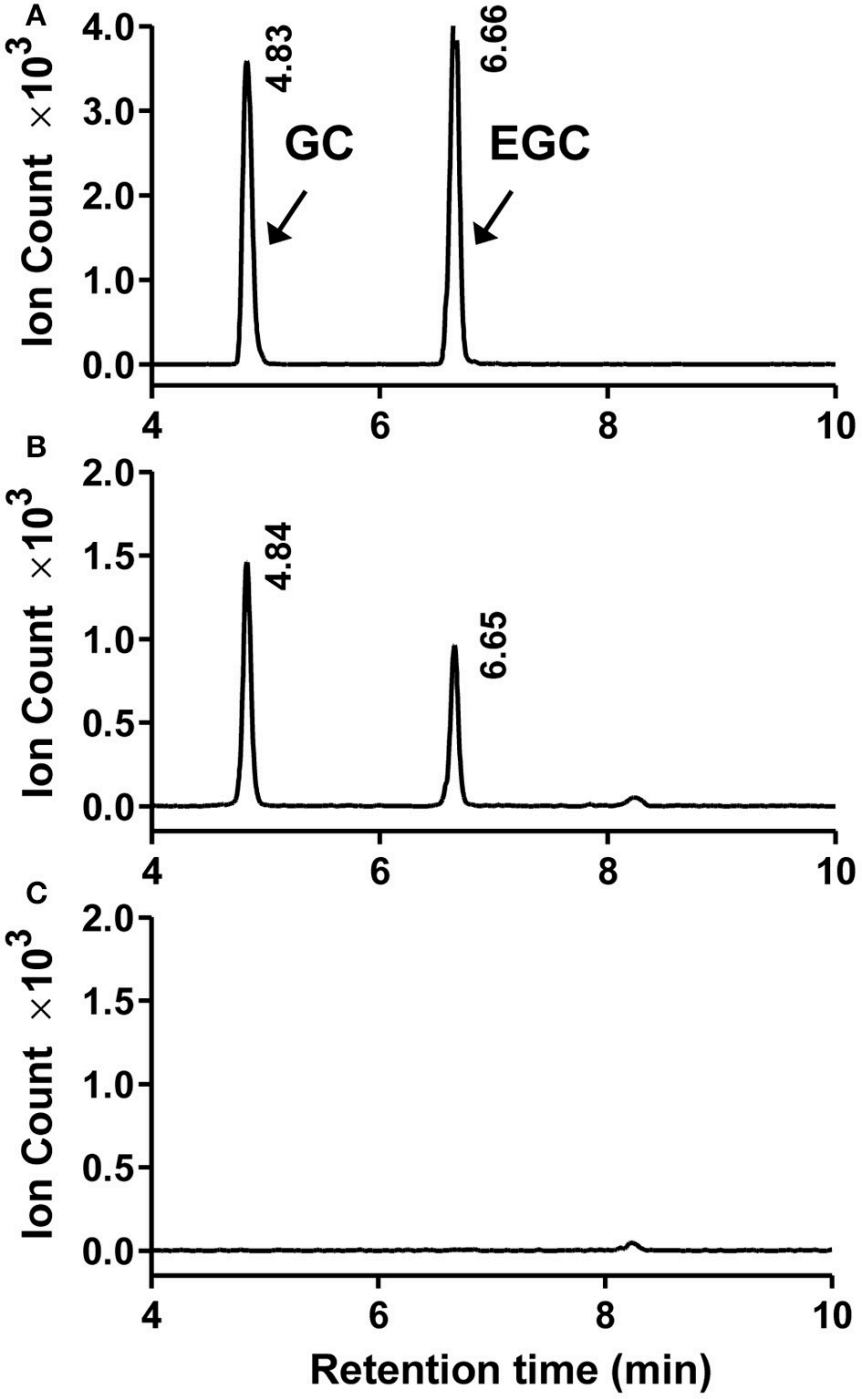
Enzymatic assay of recombinant MrANR using delphinidin as a substrate. **(A–C)**. LC-MS [M-H]^−^ extracted ion chromatographs (*m*/*z* = 305) of authentic (+)-gallocatechin (GC) and (−)-epigallocatechin (EGC) **(A)** along with the enzymatic products of purified recombinant MrANR protein **(B)** or protein extract from vector control **(C)**.

To determine the catalytic activity of *MrANR*, the *MrANR* ORF was subcloned into the bacterial expression vector pET32a, expressed in *Escherichia coli* and purified. Denaturing gel electrophoresis revealed that the recombinant MrANR protein had a molecular mass of approximately 56 kDa, which most likely corresponded to the 20 kDa of Trx.Tag-His.Tag-S.Tag sequences (Kurup et al., 2000) fused to the expected 36 kDa *MrANR* gene product (Figure 4A). Based on the bayberry PA subunit composition data, cyanidin and delphinidin were the primary *in planta* substrates for formation of EC and EGC as well as their gallate esters, ECG and EGCG, respectively. However, no epiafzelechin (EAZ; with pelargonidin as a substrate) was detected in Chinese bayberry fruit. Therefore, recombinant MrANR enzyme was assayed using cyanidin and delphinidin as substrates. When the enzymatic products were analyzed by LC-MS/MS, their chromatograms and ions spectra were identical with those of the corresponding authentic *cis*-flavan-3-ol standards, EC and EGC (Figures 4B,C, 5A,B and Figure S3A). Interestingly, the *trans*-flavan-3-ol products with the same chromatographic and MS/MS patterns as C and gallocatechin (GC) were also detected when MrANR protein was incubated with cyanidin and delphinidin, respectively. No products were generated in incubations with protein from the vector control (Figures 4D, 5C).

**Figure 6:**
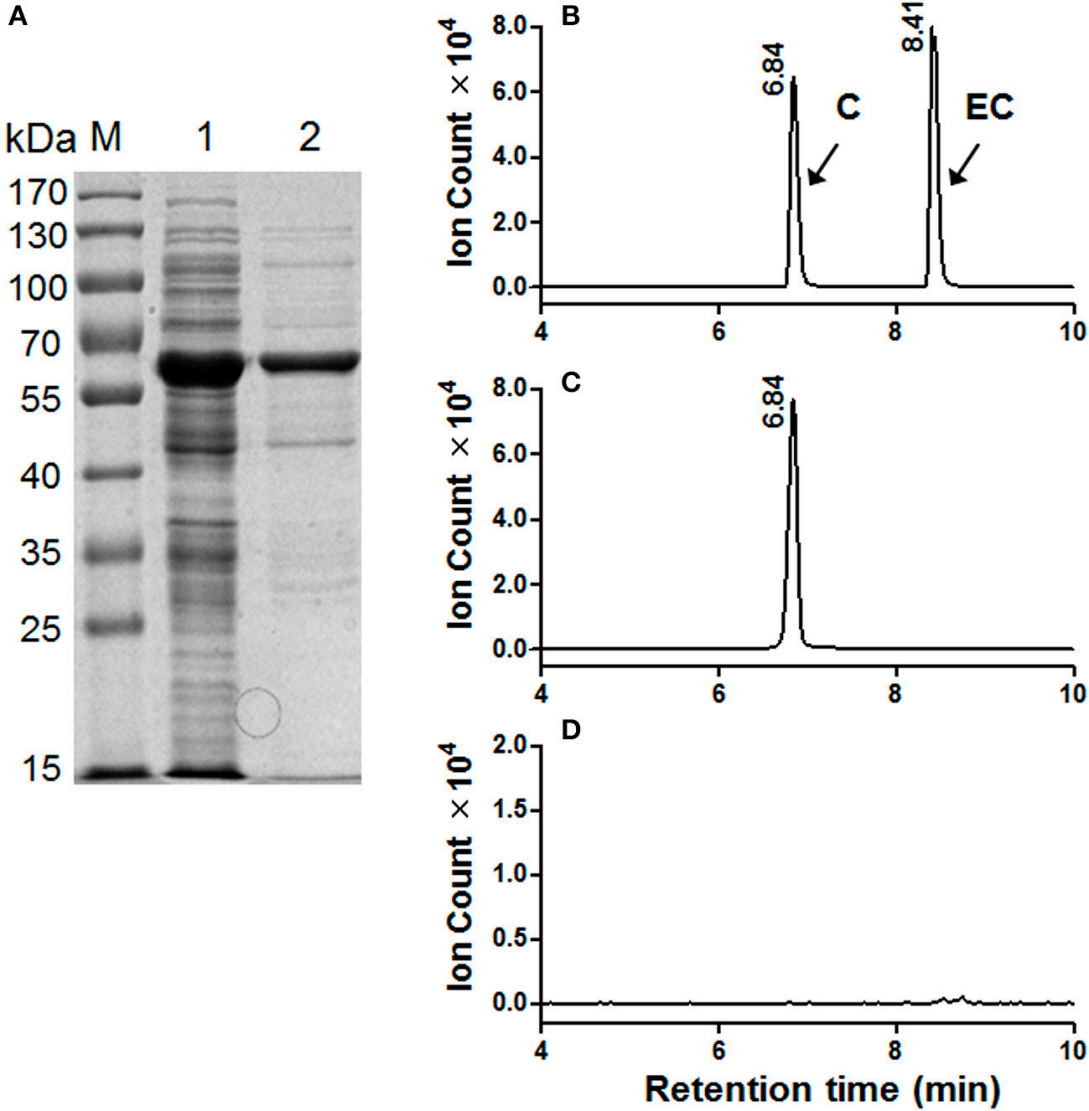
Enzymatic assay of recombinant MrLAR using leucocyanidin as a substrate. **(A)** Analysis of MrLAR protein on a 12.5% SDS-PAGE gel. M, Molecular weight marker; 1, crude protein from *E. coli* BL21 (DE3) harboring pET32α-MrLAR induced by IPTG; 2, purified recombinant MrLAR protein. **(B–D)**. LC-MS [M-H]^−^ extracted ion chromatographs (*m*/*z* = 289) of authentic (+)-catechin **(C)** and (−)-epicatechin (EC) **(B)** along with the enzymatic products of purified recombinant MrLAR protein **(C)** or protein extract from vector control **(D)**.

The MrLAR ORF was expressed as an N-terminal 6 × His tagged recombinant protein in *Escherichia coli*. The purified recombinant protein showed a single protein band of around 59 kDa, higher than its predicted molecular mass (39 kDa), which can be ascribed to its N-terminal fusion of Trx.Tag-His.Tag-S.Tag sequences (about 20 kDa) (Figure 6A). Because C was the predominant *trans*-flavan-3-ol in Chinese bayberry fruits, the MrLAR catalytic activity for the conversion of leucocyanidin to C was analyzed by LC-MS/MS in comparison to the authentic standards. The LC-MS/MS chromatograms and ions spectra confirmed the formation of C, whereas no EC product was detected (the retention time of 8.41 min) (Figures 6B,C and Figure S3B). Control incubations with protein from the vector control gave no conversion of leucocyanidin (Figure 6D).

### Ectopic expression of *MrANR* in tobacco

**Figure 7:**
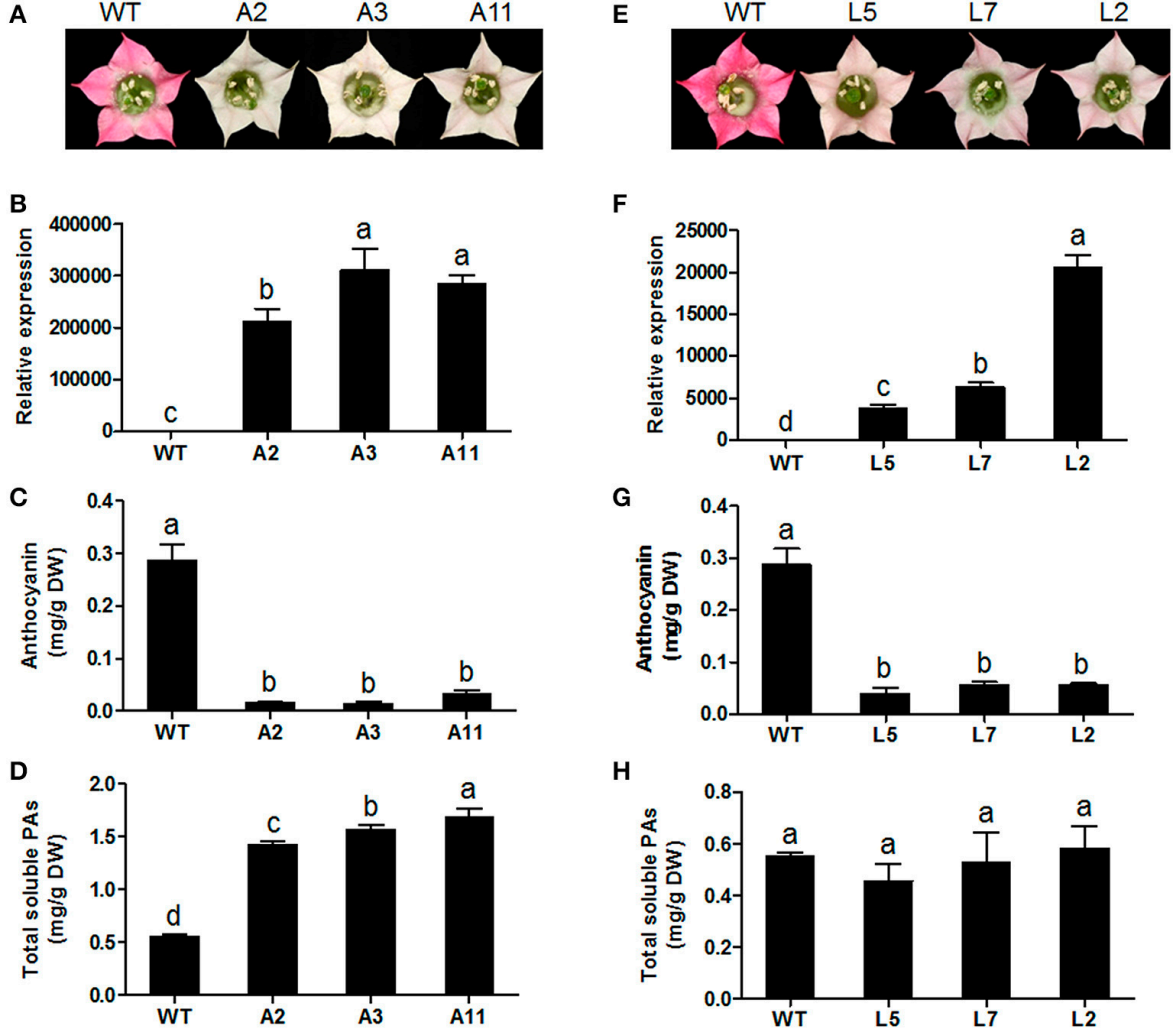
Overexpression of *MrANR* and *MrLAR* in tobacco. **(A)** Flower color of wild type (WT) and three independent lines of *MrANR* transgenic (A2, A3 and A11,) tobacco plants. **(B)** Expression profiles of *MrANR* in transgenic flowers. **(C)** Anthocyanin levels in flowers of WT and *MrANR* transgenic plants. **(D)** Total soluble PAs levels in flowers of WT and *MrANR* transgenic plants. **(E)** Flower color of wild type (WT) and three independent lines of *MrLAR* transgenic (L5, L7 and L2) tobacco plants. **(F)** Expression proflles of *MrLAR* in flowers of transgenic tobacco lines. **(G)** Anthocyanin levels in flowers of plants shown in **(E)**. **(H)**, Total soluble PAs levels in flowers of of plants shown in **(E)**. All data are presented as mean ± SD of three replicate reactions. Different letters above the bars indicate significant difference between wild-type and transgenic plants (*P* < 0.05 by Duncan's multiple range test).

To characterize the *in vivo* function of MrANR, its ORF was ectopically expressed in tobacco (cv. Samsun) driven by the cauliflower mosaic virus (CaMV) 35S promoter. From 25 independent hygromycin-resistant transgenic tobacco lines, nine plants exhibited a decrease in pink color intensity in flower petals. Three independent lines (Lines A2, A3, and A11) displaying virtually white petals were selected for further analysis (Figure 7A and Figure S4A). The qRT-PCR analysis confirmed extremely high levels of *MrANR* transcripts in these three lines (Figure 7B). Quantification of anthocyanin and soluble PA levels indicated that anthocyanin levels of all three lines were significantly lower than those of wild-type (WT) flowers. On the contrary, their soluble PA contents were significantly higher (Figures 7C,D). Insoluble PA levels were also determined, but with no significant difference between WT and transgenic plants (Figure S5A). Thus, in these lines, an inverse relationship between the level of anthocyanins and PAs was detected. The structure of PAs in the transgenic tobacco lines was further confirmed by using phloroglucinolysis and LC-MS/MS analyses (Table 1 and Figure S6A). Compared with the WT, overexpression of *MrANR* in tobacco resulted in a significant increase in the concentration of all PA subunits, including catechin-terminal units and epicatechin-terminal and extension units. Moreover, an increased mDP ranging from 13.2 to 16.4 subunits was also observed in the overexpressing lines (Table 1).

**Table 1:** PA composition and content (mg/g DW) in wild-type and *MrANR* transgenic tobacco flowers.

**Tobacco**	**Catechin in terminal units**	**Epicatechin in terminal units**	**Epicatechin in extension units**	**mDP**
WT	1.45 ± 0.02d	1.97 ± 0.10d	27.48 ± 0.07c	8.8 ± 0.42c
A2	1.88 ± 0.07c	2.92 ± 0.09c	74.00 ± 0.89b	16.4 ± 0.33a
A3	2.36 ± 0.03b	3.46 ± 0.10b	88.01 ± 0.24a	16.1 ± 0.23a
A11	3.02 ± 0.18a	4.58 ± 0.51a	89.91 ± 0.41a	13.2 ± 0.68b

*All data correspond to mean values±SD of three biological replicates*.

*Different letters in each data column indicate significant difference between wild-type and transgenic plants (P < 0.05 by Duncan's multiple range test)*.

*PA content was determined using the phloroglucinolysis and LC-MS/MS analysis method*.

**Figure 8:**
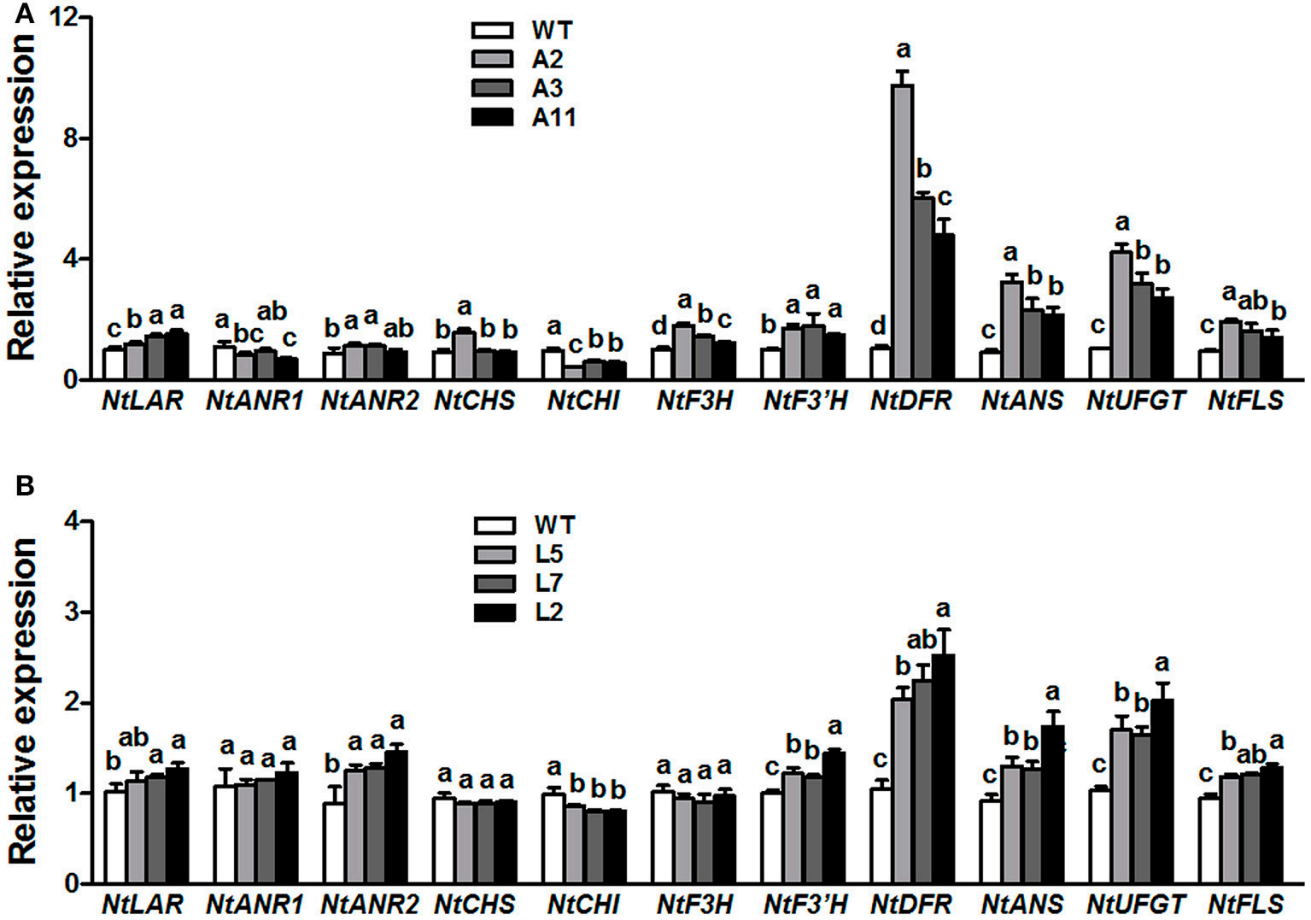
Expression profiles of flavonoid-related structural biosynthetic genes in flowers of *MrANR* **(A)** and *MrLAR* **(B)** transgenic tobacco lines. All data are presented as mean ± SD of three replicate reactions. Different letters above the bars indicate significant difference between wild-type and transgenic plants (*P* < 0.05 by Duncan's multiple range test).

QRT-PCR analysis was conducted to elucidate the effect of ectopic expression of *MrANR* on flavonoids pathway in tobacco flowers. The results showed that overexpressing *MrANR* strongly affected expression of flavonoid structural genes in transgenic tobacco flowers (Figure 8A). Most of the structural genes, including *NtLAR, NtANR2, NtF3H, NtF3'H, NtDFR, NtANS, NtUFGT*, and *NtFLS*, were up-regulated in flowers of all three *MrANR* overexpression lines. However, transcripts of *NtCHI* in the three lines were significantly lower than in WT.

### Ectopic expression of *MrLAR* in tobacco

Thirty independent hygromycin-resistant transgenic tobacco lines were generated after introduced with the ORF of *MrLAR*. Among 10 plants with visibly decreased color, three lines (Lines L5, L7 and L2) with the greatest decrease in petal color (Figure 7E and Figure S4B) and high levels of *MrLAR* transcripts (Figure 7F) were selected for further analysis. Compared with WT, these three lines exhibited a strong reduction in anthocyanin content (Figure 7G). Surprisingly, no significant difference in soluble nor insoluble PA levels was observed between *MrLAR* transgenic plants and WT flowers (Figure 7H and Figure S5B). However, all of L5, L7 and L2 lines accumulated higher levels of catechin-terminal units known as the predicted product of MrLAR, and epicatechin-terminal units but lower epicatechin-extension units than WT (Table 2 and Figure S6B). Moreover, the mDP was about 2 to 5 times lower in these transgenic lines than that in WT (Table 2). All three transgenic plants displayed significantly higher transcript abundance of most flavonoid structural genes, including *NtANR2, NtF3'H, NtDFR, NtANS, NtUFGT*, and *NtFLS* but lower *NtCHI* as compared to WT (Figure 8B). *In vivo* genetic analysis also showed that overexpression of *MrANR* and *MrLAR* led to accumulation of PAs in tobacco leaves, but changes in total soluble PAs and expression of PA-related genes in leaves of transgenic lines compared to wild type were much less marked than those in flowers (Figure S7).

**Table 2:** PA composition and content (mg/g DW) in wild-type and *MrLAR* transgenic tobacco flowers.

**Tobacco**	**Catechin in terminal units**	**Epicatechin in terminal units**	**Epicatechin in extension units**	**mDP**
WT	1.37 ± 0.23c	1.90 ± 0.08c	23.48 ± 0.11a	8.3 ± 0.34a
L5	6.07 ± 0.61b	3.89 ± 0.72b	18.44 ± 0.57c	2.8 ± 0.18b
L7	5.59 ± 0.16b	3.52 ± 0.26b	19.91 ± 0.43b	3.2 ± 0.06b
L2	10.75 ± 0.30a	6.86 ± 0.26a	14.32 ± 0.91d	1.8 ± 0.01c

*All data correspond to mean values±SD of three biological replicates*.

*Different letters in each data column indicate significant difference between wild-type and transgenic plants (P < 0.05 by Duncan's multiple range test)*.

*PA content was determined using the phloroglucinolysis and LC-MS/MS analysis method*.

## Discussion

PAs, the products of the flavonoid pathway, are abundant in many fruits, with a strong impact on their taste and health benefits. PA concentration has been found to decrease in the progression of ripening in a variety of fruits, such as bilberry (Jaakola et al., 2002), grape (Bogs et al., 2005), and strawberry (Carbone et al., 2009). Interestingly, soluble PAs in Chinese bayberry fruit also declined as the fruit matured while an increase in insoluble PAs levels was observed in the late stages of fruit development (Figures 2B,C), which suggested that the PAs in bayberries are initially soluble (potentially in the cell vacuole) and are subsequently insolubilized (presumably in the cell wall) (Pang et al., 2007). *ANR* and *LAR* genes are known to encode the key enzymes involved in PA biosynthesis, and both exhibit down-regulation pattern during ripening in many fruits, which is consistent with the decrease in soluble PA levels (Jaakola et al., 2002; Bogs et al., 2005; Carbone et al., 2009). In present study, high transcript level of *MrANR* was observed at early stages of fruit development, which was correlated with the early accumulation of soluble PAs, indicating the possibility of the early involvement of *MrANR* in PA accumulation in bayberries. In contrast, the up-regulated expression of both *MrANR* and *MrLAR* genes during the later stages of ripening was well associated with the increase in insoluble PA content. Therefore, these results suggested that both MrANR and MrLAR may play an important role in PA synthesis with a temporal-specific manner in Chinese bayberry fruit.

The soluble PA oligomers in Chinese bayberry fruit were demonstrated to contain EGCG as the predominant terminal subunit, while EGCG, EGC and C were the major components of the extension subunit (Figures 3A,B). This result is agreeable with previous studies showing that PAs in Chinese bayberry leaves were composed of EGCG as their terminal subunit and most of their extension subunits (Yang et al., 2011; Fu et al., 2014). In contrast, the PAs of grapes, blueberry, strawberry, and other berries contain a high proportion of EGC, whereas galloylated EGC (EGCG) are undetectable (Cohen et al., 2012; Huang et al., 2012). The presence of galloyl groups was reported to potentially enhance the antioxidative activity of PAs (Aron and Kennedy, 2008). ANR has been characterized as producing cis-flavan-3-ols (e.g., EGCG or EGC) in a number of species (Xie et al., 2003; Bogs et al., 2005; Akagi et al., 2009), while LAR producing trans-flavan-3-ols (e.g., C) (Tanner et al., 2003). Our enzymatic analysis showed that recombinant MrANR protein use cyanidin and delphinidin as substrates to produce corresponding EC and EGC. For LAR activity, *in vitro* experiments suggested that the LAR enzymes characterized from Chinese bayberry efficiently catalyze the production of C from leucocyanidin (Figure 6). Therefore, in this study, the reduction of soluble PA in bayberries was due to the decline in all cis-flavan-3-ol terminal and extension subunits during the earlier stages of bayberry development, which was closely related to the expression pattern of *MrANR*. On the contrary, the increase in C extension subunits in late stages was correlated with the up-regulated expression of *MrLAR*. Intriguingly, in addition to EC and EGC, the MrANR protein in our study also produced C and GC from the substrates of cyanidin and delphinidin, respectively, suggesting that the MrANR protein may have an epimerase activity *in vitro*. Such epimerase activity inherently associated with ANR has been reported in *Gossypium hirsutum* (Zhu et al., 2015), *Vitis bellula* (Zhu et al., 2014), *Vitis vinifere* (Gargouri et al., 2010) and *Camellia sinensis* (Pang et al., 2013), producing cis-flavan-3-ols *in vitro* as well as trans-flavan-3-ols.

The functionality of *MrANR* and *MrLAR* was further investigated by ectopic expression in tobacco. Over-expression of *MrANR* in tobacco led to a reduction of anthocyanidin level but an increase in C-terminal unit including EC-terminal and extension unit. Our results were similar to previous reports that over-expression of the *ANR* gene in tobacco diverted the metabolic flow from anthocyanin to PA synthesis (Xie et al., 2003; Liu et al., 2013; Pang et al., 2013). Likewise, ectopic expression of *MrLAR* resulted in a decrease in the levels of anthocyanin pigments in tobacco flowers as well. Surprisingly, no significant difference in PA levels was observed between *MrLAR* transgenic plants and WT flowers, which were probably associated with the elevated levels of both C and EC terminal units together with a corresponding decrease of EC-extension unit in transgenic tobacco flowers compared to WT (Table 2). Similar results were also found in PAP1-expressing tobacco when introduced with tea *LAR* gene (Pang et al., 2013). PA accumulation was even reduced in transgenic tobacco overexpressed the *MdLAR1* gene (Liao et al., 2015). Furthermore, the opposite effects of *MrANR* and *MrLAR* overexpression on EC-extension unit accumulation seemed to cause the adverse change in mDP between transgenic and WT plants, with mDP being increased in *MrANR* transgenic tobacco but decreased in *MrLAR* transgenic tobacco. Taken together, these results suggested that the ectopic expression of *MrANR* or *MrLAR* could affect the PA properties in tobacco including PA concentration, composition and polymerization.

CHI is a key enzyme involved in anthocyanin metabolism and silencing of *CHI* gene in tobacco is highly effective in reduction of anthocyanin accumulation (Nishihara et al., 2005). It was reported that overexpression of *CsANR, MdANR* or *MdLAR1* in tobacco suppressed expression of *CHI* gene in flowers, resulting in a loss of anthocyanin (Han et al., 2012; Kumar and Yadav, 2012; Liao et al., 2015). Consistently, ectopic expression of *MrANR* and *MrLAR* in present study also inhibited expression of *CHI* gene in tobacco flowers, which were related to the decrease in anthocyanin accumulation. In contrast, the expression of multiple other flavonoid structural genes, such as *NtF3'H, NtDFR, NtANS, NtUFGT*, and *NtFLS*, was up-regulated in flowers of transgenic plants carrying either *MrANR* or *MrLAR* (Figure 8). The mechanism by which these genes regulated the changes of anthocyanin and PA accumulation in transgenic tobacco is still unclear, but similar results were also reported in previous findings. For example, the increased transcript levels of *NtF3'H* and *NtUFGT* genes were observed in flowers of transgenic tobacco plants overexpressed *MdANR1* or *MdANR2a* (Han et al., 2012). Overexpression of *CsANR* enhanced the expression levels of *NtFLS* (Kumar and Yadav, 2012).

In summary, we successfully isolated two genes encoding key PA biosynthetic enzymes in Chinese bayberry fruit, *MrANR* and *MrLAR*. Their expression pattern coincided with the change of soluble and insoluble PA accumulation in developing bayberry fruit. *In vitro* enzyme assays of MrANR and MrLAR recombinant protein verified their predicted function in PA biosynthesis. Moreover, *in vivo* genetic analysis of *MrANR* in tobacco demonstrated its role in promoting the PA biosynthesis pathway. Although overexpression of *MrLAR* in tobacco was unable to affect the total PA contents, an increase in catechin levels was observed in flowers of transgenic plants. Therefore, we concluded that *MrANR* and *MrLAR* were responsible for the biosynthesis of flavan-3-ol monomers in Chinese bayberry fruit to some extent. In plants, PAs are colorless flavonoid polymers formed by polymerization of flavan-3-ol units with sequential addition of extension subunits to a flavan-3-ol terminal unit. It has been reported recently that excessive 4β-(*S*-cysteinyl)-epicatechin, which is a carbocation form, played an important role in non-enzymatic polymerization and served as a PA extension unit (Liu et al., 2016). LARs could catalyze it to form EC *in vitro* and consequently inhibited the polymerization of PAs (Liu et al., 2016; Wang et al., 2018). Thus, the biosynthesis of carbocation and its effect on PA polymerization in bayberries should be investigated further to reveal the true role of *MrLAR*.

## Author contributions

ZY and YZ conceived and designed the study. LS, SC, XC, and WC performed experiments, analyzed and interpreted the data, and wrote the manuscript. LS, SC, ZY, and YZ revised the manuscript critically. All authors read and approved the final manuscript.

### Conflict of interest statement

The authors declare that the research was conducted in the absence of any commercial or financial relationships that could be construed as a potential conflict of interest.
