# Supplementary material for: Proanthocyanidin Synthesis in Chinese Bayberry (Myrica rubra Sieb. et Zucc.) Fruits
Source: Front Plant Sci. 2018 Feb 28;9:212. doi: 10.3389/fpls.2018.00212 (PMC5835688; doi:10.3389/fpls.2018.00212)
Supplement: Supplementary file 1 [file Table1.DOC]

***Supplementary Material***

**Proanthocyanidin synthesis in Chinese bayberry (*Myrica rubra* Sieb. et Zucc.) fruits**

Liyu Shi 1, Shifeng Cao 2, Xin Chen 2, Wei Chen 2, Yonghua Zheng 1,*, and Zhenfeng Yang 2,*

*** Correspondence:** zhengyh@njau.edu.cn & yangzf@zwu.edu.cn

**Table S1** Primers used in this study

| **Primer names^a^** | **Sequence (5' to 3')** |
| --- | --- |
| MrANR degenerate F | CATCTCAAGATCCTGAAAATGATATGATHAARCC |
| MrANR degenerate R | CAAAAATATGAGCTCGACAAACATCNTCNACRTG |
| MrANR RACE 5’-gsp | CATGGCTAGACCAAGACAGCTCGGGACA |
| MrANR RACE 5’-nested | GAGCAGGACCGCCCATAAGACTAGGAATCACA |
| MrANR RACE 3’-gsp | CTTGGGGTTATCCTGCTTC |
| MrANR RACE 3’-nested | CCAGATGTCCCGAGCTGTCTTG |
| MrANR-F | CGACGTTGGATTCCTAAGAT |
| MrANR-R | AGGCAACAGCACATCTCCCAGT |
| MrLAR degenerate F | GACATGATGTTGATAGAGCTGATCCNGTNGARCC |
| MrLAR degenerate R | AAAATTAACTTGACATCCCTTAATAAAAATATCRTGNGTRAA |
| MrLAR RACE 5’-gsp | TCGGACGGGTGGGTGTTGTCGTAGTAG |
| MrLAR RACE 5’-nested | GACTCCTCCACCAGCCGCCTCACT |
| MrLAR RACE 3’-gsp | ACAACACCCACCCGTCCGAG |
| MrLAR RACE 3’-nested | AGTGTTCATTTTCGACCTACCTG |
| MrLAR-F | CAAAACAGCCTCGTCAAACT |
| MrLAR-R | TTGGTTGAATAAGGAAATCACTTG |
| MrANR-BF | GGATCCATGGCCACCCAACACCTTG |
| MrANR-SR | GTCGACTCACTTCTGCAGCAGGCCCTT |
| MrLAR-BF | GGATCCATGACCGTGACACCTATTTC |
| MrLAR-SR | GTCGACTCAAGCACATGTTGCAGTGAT |
| MrANR qRT-PCR F | TGCCGTCAATACCACTGTAAGAG |
| MrANR qRT-PCR R | CGATATGGAACACAAGGTCACAAC |
| MrLAR qRT-PCR F | CAGAACGAAGAGGAGAGTG |
| MrLAR qRT-PCR R | TCCAGCAACGAAGTAAGC |
| MrACT qRT-PCR F | AATGGAACTGGAATGGTCAAGG |
| MrACT qRT-PCR R | CCCGACATAGGCATCTTTCTG |
| NtCHS qRT-PCR F | TCGGTCAAGCGGTTCAT |
| NtCHS qRT-PCR R | GTCATTGGGTCCACGAAAC |
| NtCHI qRT-PCR F | ATTGAAGGGAAGTTTGTGAAGT |
| NtCHI qRT-PCR R | ACCCGTCAAAGGCAAGA |
| NtF3H qRT-PCR F | CCAGATGGATGGATAGGTG |
| NtF3H qRT-PCR R | GGTAAGGTCGGGCTGTG |
| NtF3'H qRT-PCR F | CGTGATGGAATCCGACCTA |
| NtF3'H qRT-PCR R | AAGTCATTTCCTCGCACATC |
| NtDFR qRT-PCR F | GCCGCTGGTTGTTGGTC |
| NtDFR qRT-PCR R | CGCAGATGAATCTTCCCTC |
| NtANS qRT-PCR F | CTTTCTATTGGGCTGGGACT |
| NtANS qRT-PCR R | ACATCAGTATGAGCTTCAACGC |
| NtUFGT qRT-PCR F | AGAAAGTTATGAAAGAGGCAGAG |
| NtUFGT qRT-PCR R | GGACCGAATCAAATAAGTGTAT |
| NtFLS qRT-PCR F | GAACTTGAAGGGAAAAGGGG |
| NtFLS qRT-PCR R | TCCCTGTAGGAGGGAGGATT |
| NtLAR qRT-PCR F | TCAAGGTCCTTTACGCCATC |
| NtLAR qRT-PCR R | ACGAACCTGCTTCTCTTTGG |
| NtANR1 qRT-PCR F | CATTTGACTTTCCCAAACGC |
| NtANR1 qRT-PCR R | ATTGGGCTTTTGAGTTGTGC |
| NtANR2 qRT-PCR F | TGTTCCCACTTGGGATGATA |
| NtANR2 qRT-PCR R | TGCACCTATACTCTGTTAGTGGC |
| NtActin qRT-PCR F | CGTTTGGACCTTGCTGG |
| NtActin qRT-PCR R | TCTGGGCAACGGAACCT |

^a^Forward and reverse primer are abbreviated as F and R; gsp, gene specific primer.
