# Supplementary material for: Proanthocyanidin Synthesis in Chinese Bayberry (Myrica rubra Sieb. et Zucc.) Fruits
Source: Front Plant Sci. 2018 Feb 28;9:212. doi: 10.3389/fpls.2018.00212 (PMC5835688; doi:10.3389/fpls.2018.00212)
Supplement: Supplementary file 2 [file Table2.DOC]

***Supplementary Material***

**Proanthocyanidin synthesis in Chinese bayberry (*Myrica rubra* Sieb. et Zucc.) fruits**

Liyu Shi 1, Shifeng Cao 2, Xin Chen 2, Wei Chen 2, Yonghua Zheng 1,*, and Zhenfeng Yang 2,*

*** Correspondence:** zhengyh@njau.edu.cn & yangzf@zwu.edu.cn

**Table S2** Characterization of PA phloroglucinolysis products from 57 DAFB Chinese bayberry fruit by LC-MS-MS

| **Compound^a^** | **t_R_ (min)^b^** | **[M - H]^- c^** | **Fragment ions** |
| --- | --- | --- | --- |
| EGC-P | 10.13 | 429 | 303, 261, 177 |
| EGCG-P | 12.12 | 581 | 429, 285, 261,177 |
| CT-P | 12.62 | 413 | 287, 261, 217, 175 |
| ECG-P | 14.72 | 565 | 413, 269, 261, 169 |
| EGC | 14.82 | 305 | 219, 137, 125 |
| EGCG | 16.64 | 457 | 305, 287, 169, 125 |
| EC | 17.5 | 289 | 245, 137 |
| ECG | 19.02 | 441 | 289, 271, 169, 125 |

^a^ EGC-P, epigallocatechin-(4β→2)-phloroglucinol; EGCG-P, epigallocatechin-3-O-gallate-(4β→2) -phloroglucinol; CT-P, catechin-(4α→2)-phloroglucinol; ECG-P, epicatechin-3-O-gallate-(4β→2) -phloroglucinol; EGC, epigallocatechin; EGCG, epigallocatechin-3-O-gallate; EC, epicatechin; ECG, epicatechin-3-O-gallate.^b^Retention time on LC-MS. ^C^MS was run in the negative mode and all the molecular ions are [M - H]^-^.
