# Supplementary material for: Proanthocyanidin Synthesis in Chinese Bayberry (Myrica rubra Sieb. et Zucc.) Fruits
Source: Front Plant Sci. 2018 Feb 28;9:212. doi: 10.3389/fpls.2018.00212 (PMC5835688; doi:10.3389/fpls.2018.00212)
Supplement: Supplementary file 3 [file DataSheet1.doc]

**SUPPLEMENTARY MATERIAL**

The Supplementary Material for this article can be found online at:

**Table S1 Primers used in this study.**

**Table S2** **Characterization of PA phloroglucinolysis products from 57 DAFB Chinese bayberry fruit by LC-MS-MS**

**Figure S1 Alignment of deduced amino acid sequences of *ANR* genes.** Sequences are from *Morella rubra* (Mr; [AIX02996.1](https://www.ncbi.nlm.nih.gov/protein/720119061?report=genbank&log$=prottop&blast_rank=1&RID=DP6HVCJG013)), *Vitis vinifera* (Vv; NP_001267885.1), *Theobroma cacao* (Tc; ADD51353.1), *[Malus domestica](https://blast.ncbi.nlm.nih.gov/Blast.cgi#alnHdr_343082718)* (Md; AEL79860.1) and *Camellia sinensis* (Cs; AEC10993.1). Identical amino acids are indicated by a black background, conservative amino acids by a dark gray background, and similar amino acids by a light gray background. The conserved Rossmann dinucleotide-binding domain motif in N-terminal sequence GXGXXA are boxed.

**Figure S2 Alignment of deduced amino acid sequences of *LAR* genes.** Sequences are from *Morella rubra* (Mr; [AIX02997.1](https://www.ncbi.nlm.nih.gov/protein/720119064?report=genbank&log$=prottop&blast_rank=1&RID=DP4Y3SCW016)), Vitis *vinifera* (Vv; NP_001267887.1), *Camellia sinensis* (Cs; AHJ11241.1), *Theobroma cacao* (Tc; XP_007046315) and *Medicago truncatula* (Mt; XP_003591830). Identical amino acids are indicated by a black background, conservative amino acids by a dark gray background, and similar amino acids by a light gray background. LAR characteristic amino acid motifs RFLP, ICCN, and THD motifs are boxed.

**Figure S3 MS/MS spectra of ANR- and LAR-products.** A, MS/MS data for ANR-products and *cis*- and *tran*-flavan-3-ol standards. B, MS/MS data for LAR-product and (+)-catechin standard.

**Figure S4 Phenotypic analysis of flowering time and seed coat in *MrANR* transgenic lines (A) and *MrLAR* transgenic lines (B) compared to wild type (WT).**

**Figure S5 Total insoluble PAs levels in flowers of wild type (WT), *MrANR* transgenic (A) and *MrLAR* transgenic (B) tobacco plants.**

**Figure S6 HPLC chromatograms (detected at 280 nm) of PA reaction products from** **flower petals of wild type (WT) and transgenic tobacco plants with the presence of excess phloroglucinol.** A, comparison of WT reaction products with *MrANR* transgenic plants (A2, A3 and A11) reaction products. B, comparison of WT reaction products with *MrLAR* transgenic plants (L5, L7 and L2) reaction products. Peaks: 1, (−)-epicatechin-phloroglucinol; 2, (+)-catechin; 3, (−)-epicatechin.

**Figure S7 Comparison of relative PA contents and relative gene expression levels between flower and leaf tissues in transgenic tobacco plants.** A, Levels of total soluble PAs are plotted relative to wild type (red dotted line at 1.0) in flowers and leaves of *MrANR* transgenic lines (A2, A3 and A11) and *MrLAR* transgenic lines (L5, L7 and L2). B, Relative expression of *MrANR* and *MrLAR* in flowers and leaves of transgenic lines. C, Expression levels of flavonoid-related structural biosynthetic genes are plotted relative to wild type (red dotted line at 1.0) in flowers and leaves of *MrANR* transgenic lines. D, Expression levels of flavonoid-related structural biosynthetic genes are plotted relative to wild type (red dotted line at 1.0) in flowers and leaves of *MrLAR* transgenic lines.
