# Supplementary material for: Proanthocyanidin Synthesis in Chinese Bayberry (Myrica rubra Sieb. et Zucc.) Fruits
Source: Front Plant Sci. 2018 Feb 28;9:212. doi: 10.3389/fpls.2018.00212 (PMC5835688; doi:10.3389/fpls.2018.00212)
Supplement: Supplementary file 4 [file Image1.PDF]

Supplementary Material

Proanthocyanidin synthesis in Chinese bayberry (*Myrica rubra* Sieb. et Zucc.) fruits

Liyu Shi [1], Shifeng Cao [2], Xin Chen [2], Wei Chen [2], Yonghua Zheng [1,*], and Zhenfeng Yang [2,*]

*** Correspondence:** zhengyh@njau.edu.cn & yangzf@zwu.edu.cn

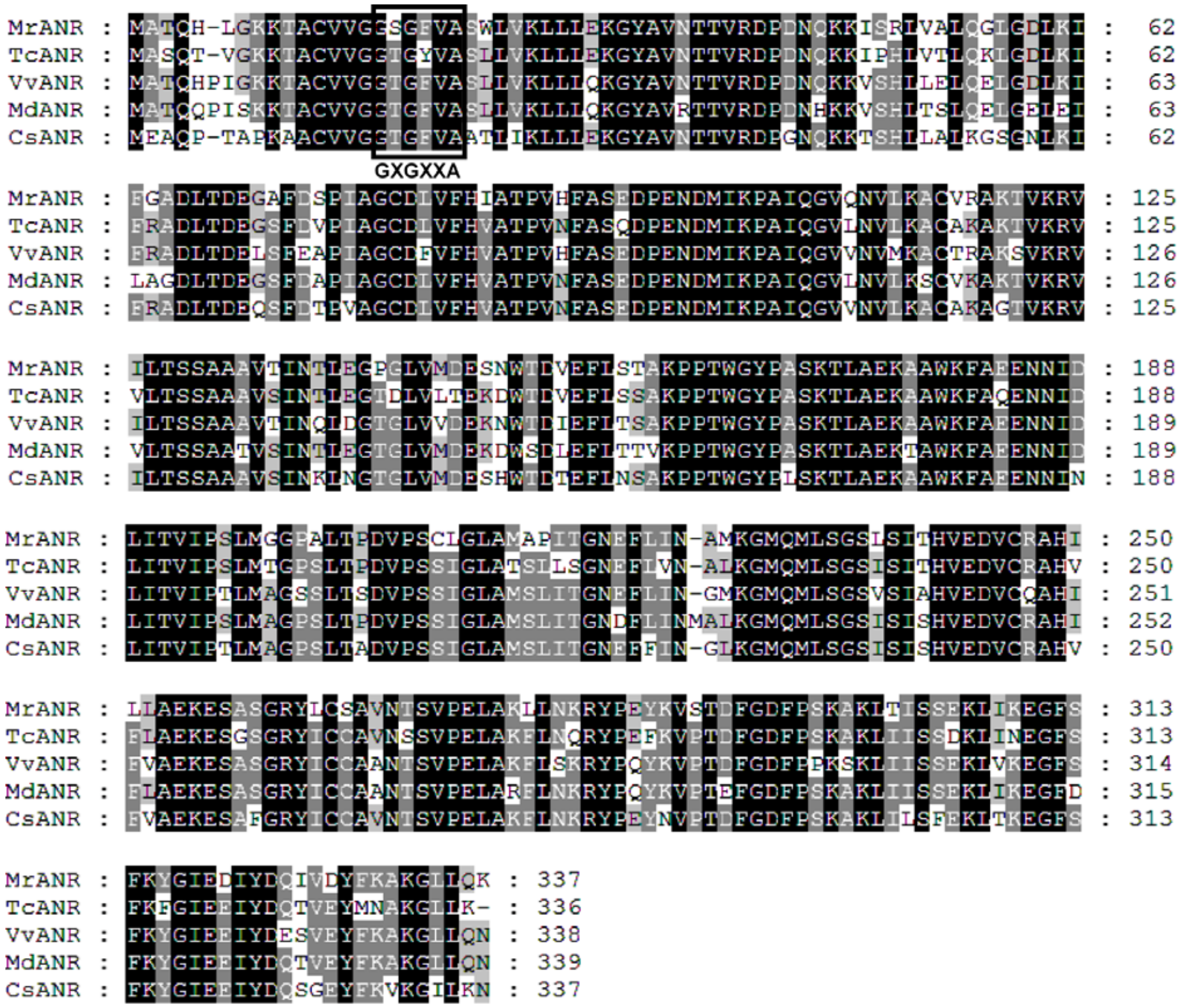

Figure S1 Alignment of deduced amino acid sequences of *ANR* genes. Sequences are from *Morella rubra* (Mr; AIX02996.1), *Vitis vinifera* (Vv; NP_001267885.1), *Theobroma cacao* (Tc; ADD51353.1), *Malus domestica* (Md; AEL79860.1) and *Camellia sinensis* (Cs; AEC10993.1). Identical amino acids are indicated by a black background, conservative amino acids by a dark gray background, and similar amino

acids by a light gray background. The conserved Rossmann dinucleotide-binding domain motif in N-terminal sequence GXGXXA are boxed.
