# Supplementary material for: Proanthocyanidin Synthesis in Chinese Bayberry (Myrica rubra Sieb. et Zucc.) Fruits
Source: Front Plant Sci. 2018 Feb 28;9:212. doi: 10.3389/fpls.2018.00212 (PMC5835688; doi:10.3389/fpls.2018.00212)
Supplement: Supplementary file 5 [file Image2.PDF]

Supplementary Material

Proanthocyanidin synthesis in Chinese bayberry (*Myrica rubra* Sieb. et Zucc.) fruits

Liyu Shi [1], Shifeng Cao [2], Xin Chen [2], Wei Chen [2], Yonghua Zheng [1,*], and Zhenfeng Yang [2,*]

*** Correspondence:** zhengyh@njau.edu.cn & yangzf@zwu.edu.cn

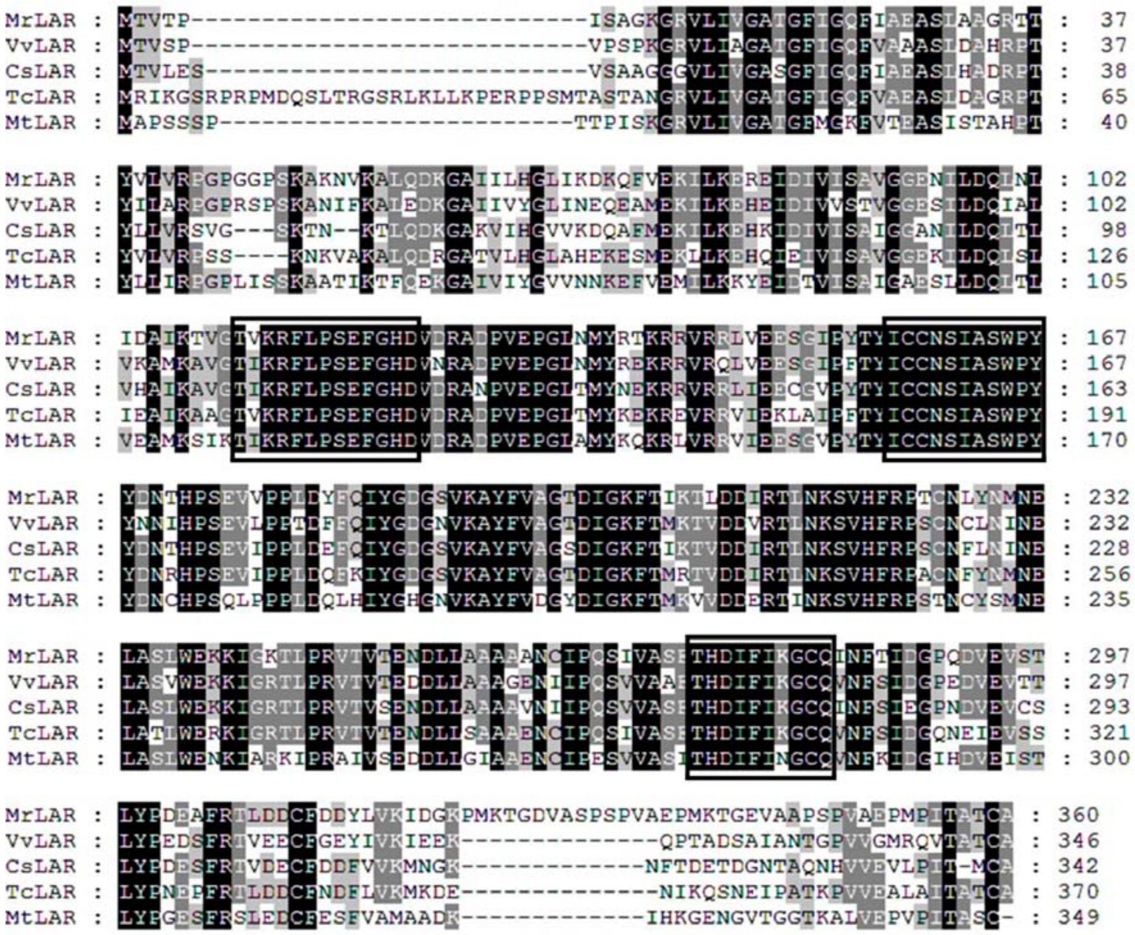

Figure S2 Alignment of deduced amino acid sequences of *LAR* genes. Sequences are from *Morella rubra* (Mr; AIX02997.1), Vitis *vinifera* (Vv; NP_001267887.1), *Camellia sinensis* (Cs; AHJ11241.1), *Theobroma cacao* (Tc; XP_007046315) and *Medicago truncatula* (Mt; XP_003591830). Identical amino acids are indicated by a black background, conservative amino acids by a dark gray background, and similar amino acids by a light gray background. LAR characteristic amino acid motifs RFLP, ICCN, and THD motifs are boxed.
