# Supplementary material for: Proanthocyanidin Synthesis in Chinese Bayberry (Myrica rubra Sieb. et Zucc.) Fruits
Source: Front Plant Sci. 2018 Feb 28;9:212. doi: 10.3389/fpls.2018.00212 (PMC5835688; doi:10.3389/fpls.2018.00212)
Supplement: Supplementary file 6 [file Image3.PDF]

Supplementary Material

Proanthocyanidin synthesis in Chinese bayberry (*Myrica rubra* Sieb. et Zucc.) fruits

Liyu Shi [1], Shifeng Cao [2], Xin Chen [2], Wei Chen [2], Yonghua Zheng [1,*], and Zhenfeng Yang [2,*]

*** Correspondence:** zhengyh@njau.edu.cn & yangzf@zwu.edu.cn

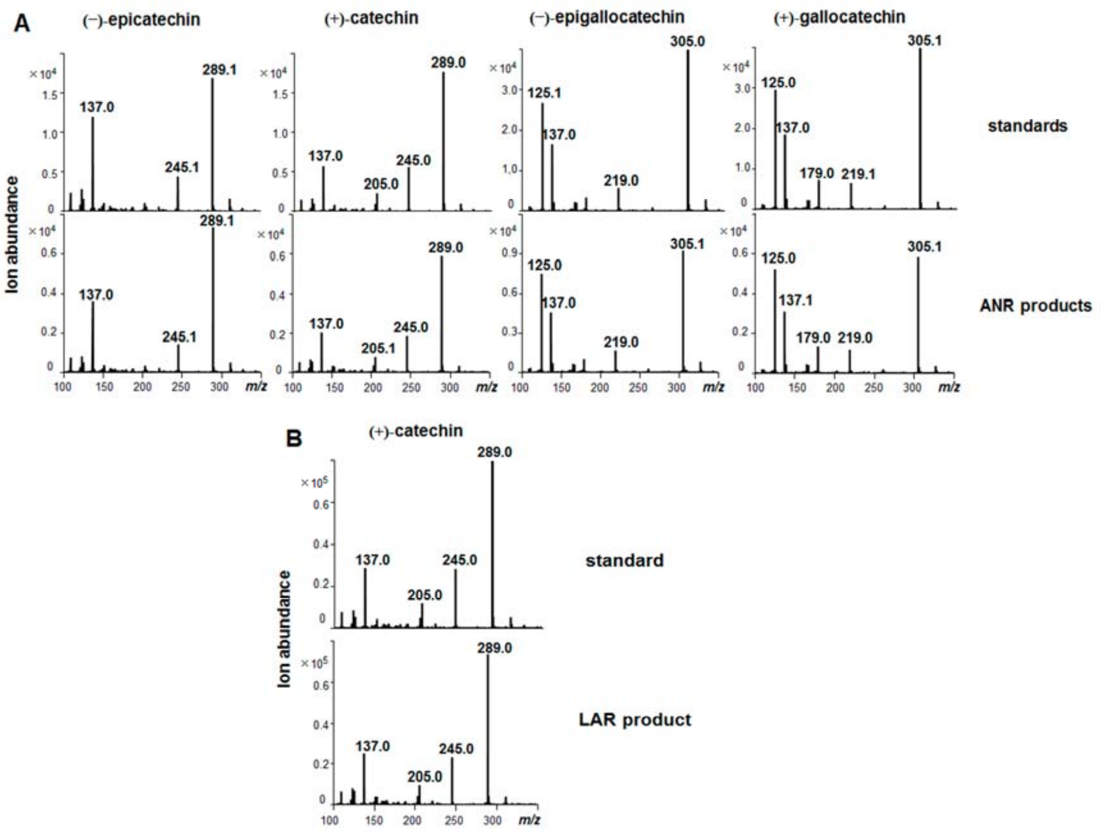

Figure S3 MS/MS spectra of ANR- and LAR-products. A, MS/MS data for ANR-products and *cis*- and *tran*-flavan-3-ol standards. B, MS/MS data for LAR-product and (+)-catechin standard.
