# Supplementary figures and images for: Proanthocyanidin Synthesis in Chinese Bayberry (Myrica rubra Sieb. et Zucc.) Fruits
Source: Front Plant Sci. 2018 Feb 28;9:212. doi: 10.3389/fpls.2018.00212 (PMC5835688; doi:10.3389/fpls.2018.00212)

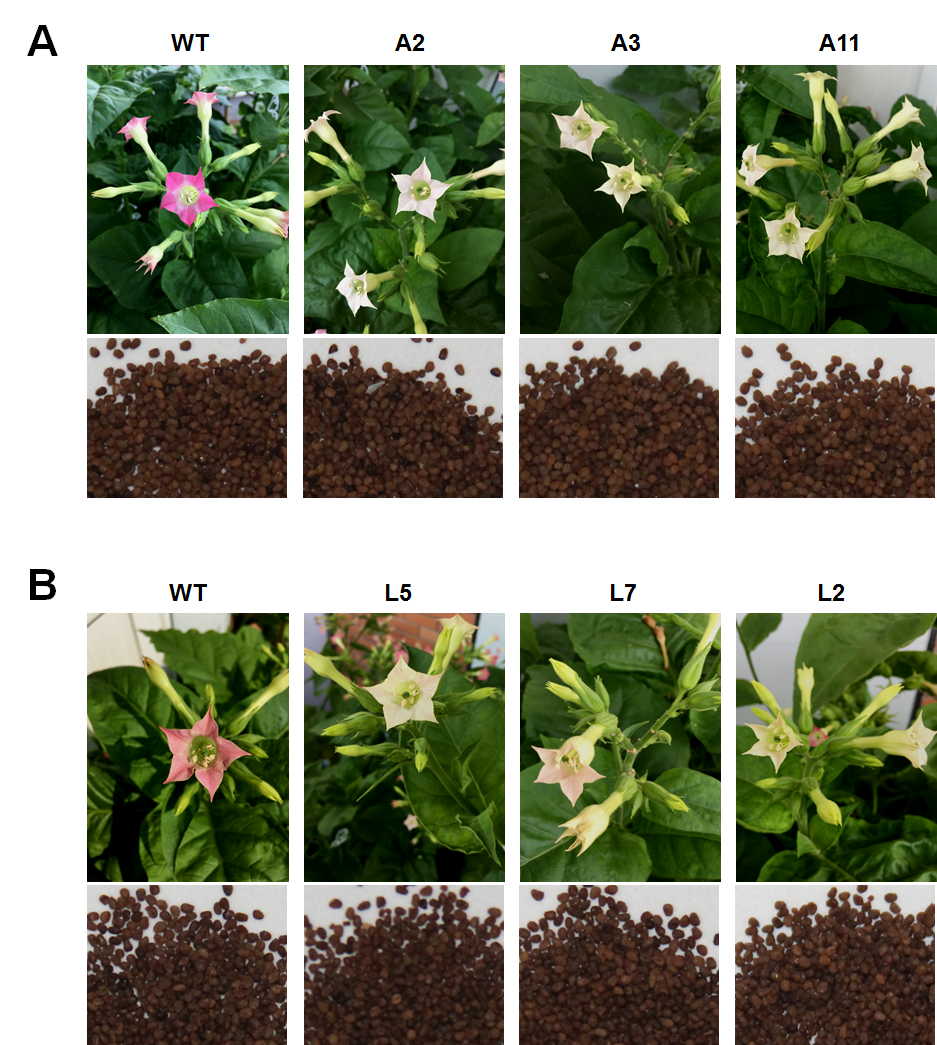

Supplement: Supplementary file 7 [file Image4.tif]

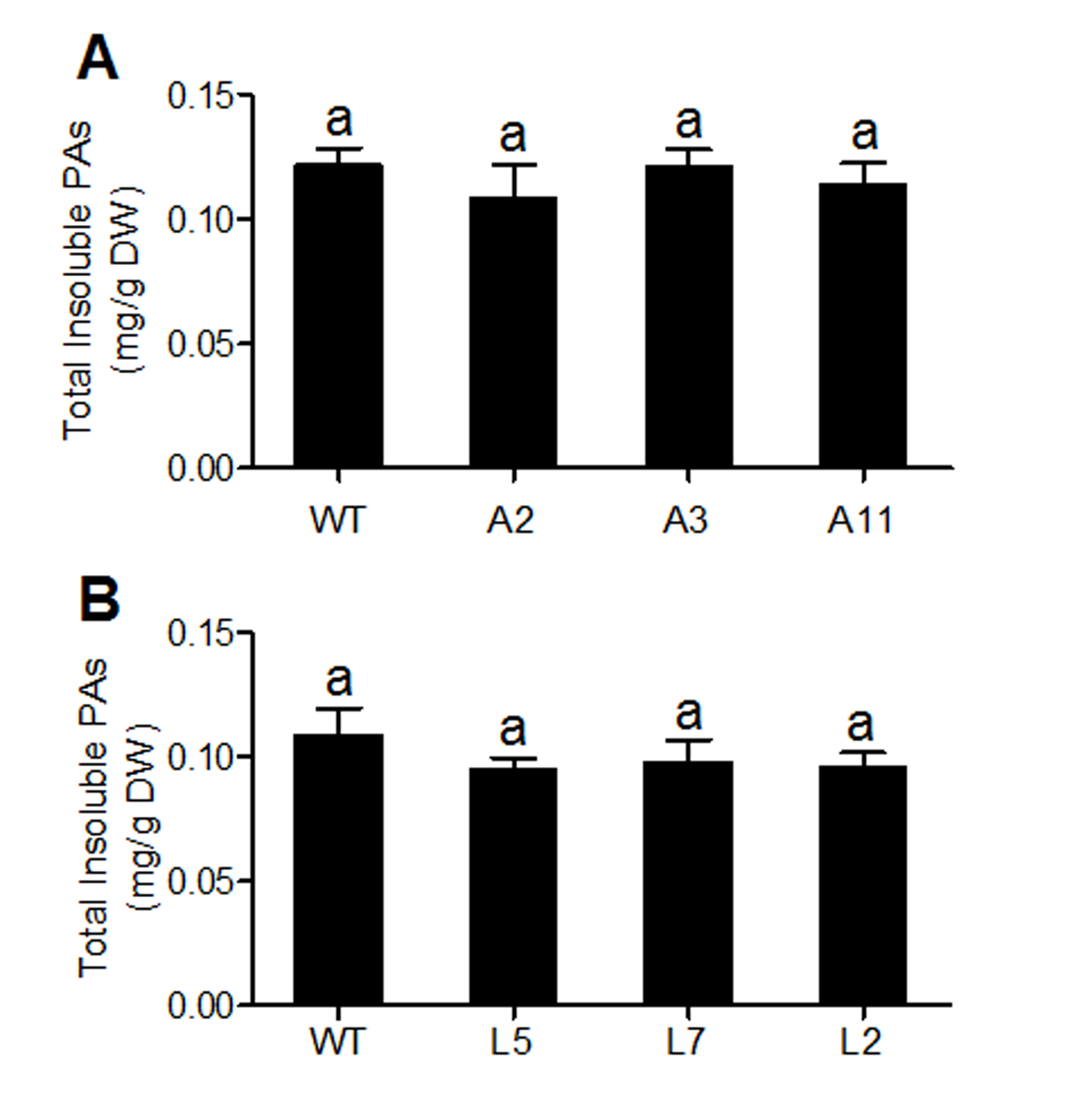

Supplement: Supplementary file 8 [file Image5.tif]

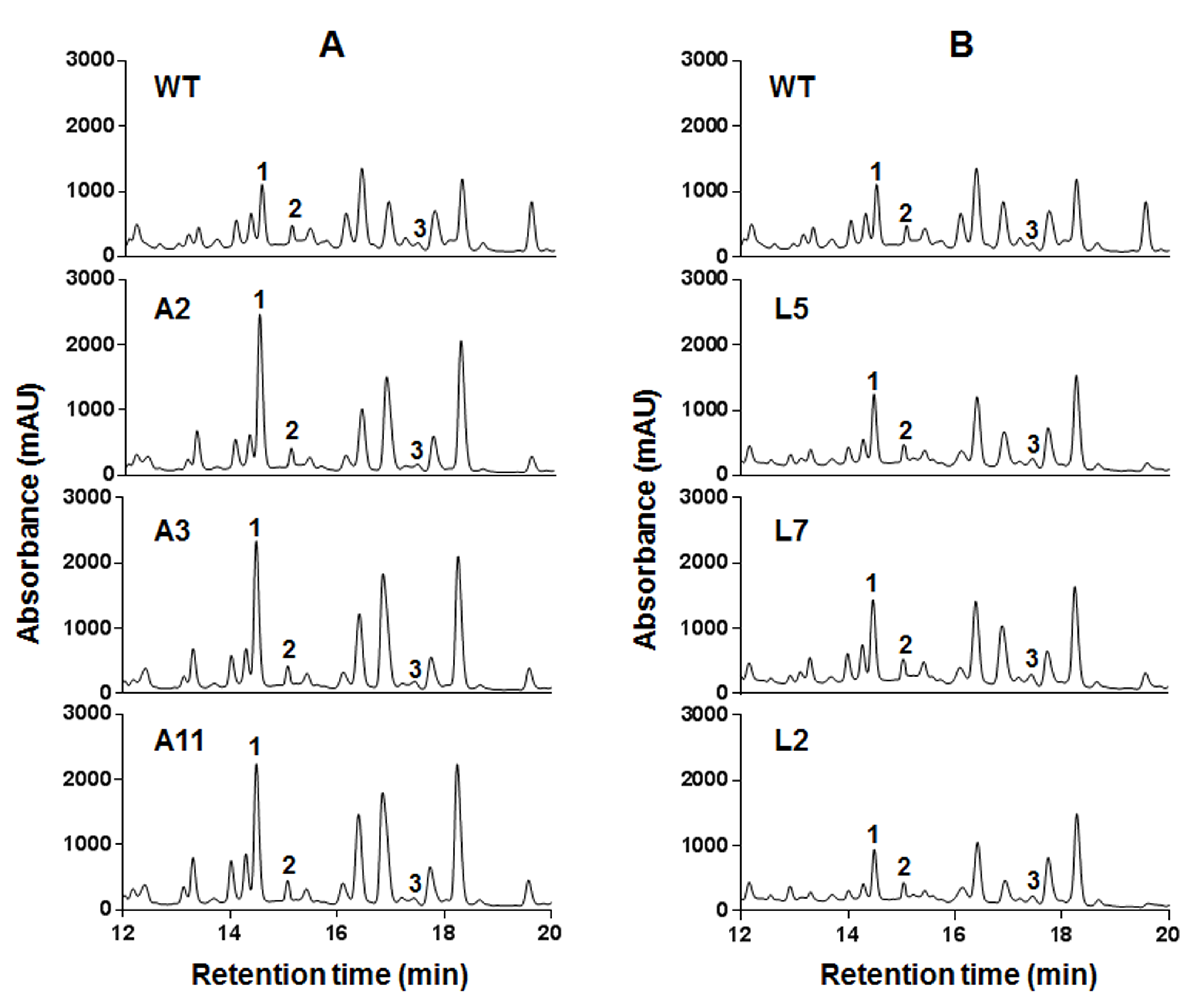

Supplement: Supplementary file 9 [file Image6.tif]
