# Supplementary material for: Proanthocyanidin Synthesis in Chinese Bayberry (Myrica rubra Sieb. et Zucc.) Fruits
Source: Front Plant Sci. 2018 Feb 28;9:212. doi: 10.3389/fpls.2018.00212 (PMC5835688; doi:10.3389/fpls.2018.00212)
Supplement: Supplementary file 10 [file Image7.PDF]

Supplementary Material

Proanthocyanidin synthesis in Chinese bayberry (*Myrica rubra* Sieb. et Zucc.) fruits

Liyu Shi [1], Shifeng Cao [2], Xin Chen [2], Wei Chen [2], Yonghua Zheng [1,*], and Zhenfeng Yang [2,*]

*** Correspondence:** zhengyh@njau.edu.cn & yangzf@zwu.edu.cn

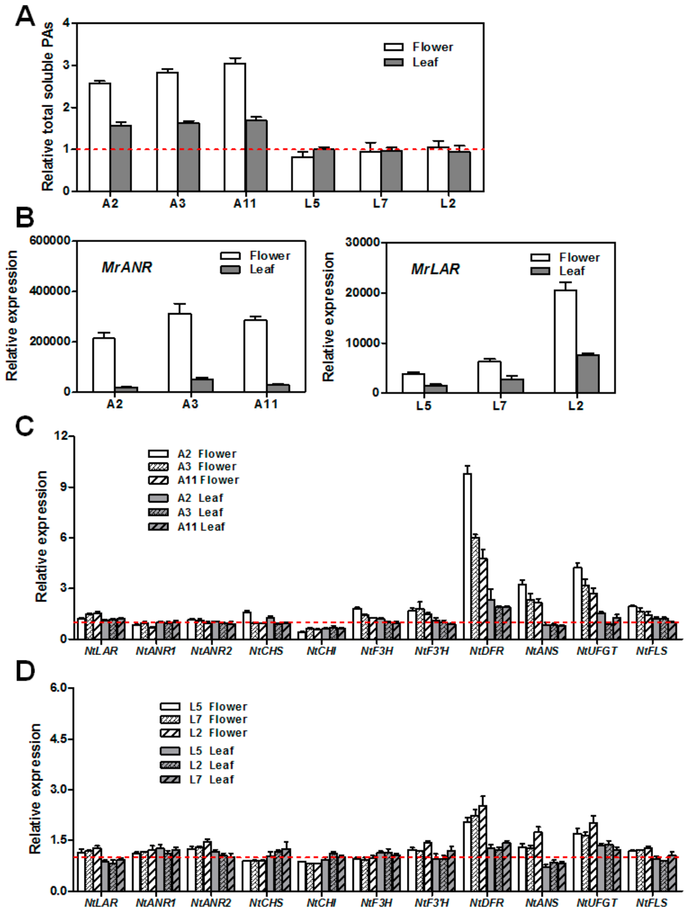

Figure S7 Comparison of relative PA contents and relative gene expression levels between flower and leaf tissues in transgenic tobacco plants. A, Levels of total soluble PAs are plotted relative to wild type (red dotted line at 1.0) in flowers and leaves of *MrANR* transgenic lines (A2, A3 and A11) and *MrLAR* transgenic lines (L5, L7 and L2). B, Relative expression of *MrANR* and *MrLAR* in flowers and leaves of transgenic lines. C, Expression levels of flavonoid-related structural biosynthetic genes are plotted relative to wild type (red dotted line at 1.0) in flowers and leaves of *MrANR* transgenic lines. D, Expression levels of flavonoid-related structural biosynthetic genes are plotted relative to wild type (red dotted line at 1.0) in flowers and leaves of *MrLAR* transgenic lines.
